# Harms and Negative or Unintended Consequences of Social Prescribing: A Scoping Review

**DOI:** 10.3390/healthcare14131947

**Published:** 2026-07-01

**Authors:** Veronika Papon, Jonas Schöpf, Jill S. Litt, Marjan Arvandi, Nerkez Opacin, Elisabeth Nöhammer, Nina Lorenzoni, Kaisu H. Pitkälä, Anu Jansson, Jan Stratil, Uwe Siebert, Ursula Rochau, Sibylle Puntscher

**Affiliations:** 1 Institute of Public Health, Medical Decision Making and Health Technology Assessment, Department of Public Health, Health Services Research and Health Technology Assessment, UMIT TIROL—University for Health Sciences and Technology, 6060 Hall in Tirol, Austria; 2 Institute for Global Health (ISGlobal), Barcelona Biomedical Research Park (PRBB), Carrer del Doctor Aiguader, 88, 08003 Barcelona, Spain; 3 CIBER Epidemiología y Salud Pública (CIBERESP), 28029 Madrid, Spain; 4 Universitat Pompeu Fabra (UPF), 08002 Barcelona, Spain; 5 School of Agriculture, Food and Ecosystem Sciences, University of Melbourne, Melbourne, VIC 3010, Australia; 6 Institute for Management and Economics in Healthcare, Department of Public Health, Health Services Research and Health Technology Assessment, UMIT TIROL—University for Health Sciences and Technology, 6060 Hall in Tirol, Austria; 7 Department of General Practice and Primary Health Care, University of Helsinki, 00130 Helsinki, Finland; 8 Unit of Primary Health Care, Helsinki University Hospital, 00029 Helsinki, Finland; 9 Miina Sillanpää Foundation, 00300 Helsinki, Finland; 10 Center for Health Decision Science, Departments of Epidemiology and Health Policy & Management, Harvard Chan School of Public Health, Boston, MA 02115, USA; 11 Institute for Technology Assessment and Department of Radiology, Massachusetts General Hospital, Harvard Medical School, Boston, MA 02114, USA

**Keywords:** social prescribing, harms, adverse events, unintended consequences, public health, community care, scoping review

## Abstract

**Background/Objectives:** Social Prescribing (SP) seeks to address non-medical needs by connecting individuals to community-based resources through a person-centered “social prescription”, such as group activities or support services. SP is increasingly being implemented internationally and has demonstrated potential benefits for wellbeing. However, potential harms and negative or unintended consequences (HNUCs) remain poorly understood. This scoping review aims to synthesize evidence on HNUCs reported in the literature. **Methods:** A systematic search of EMBASE, MEDLINE, and APA PsycInfo was conducted through September 2024 to identify full-text studies published in English or German that report HNUCs associated with SP for adults in health- or social care settings. Backward reference searches of relevant reviews identified additional studies. Two reviewers screened and extracted data independently, with disagreements resolved by a third reviewer. Study characteristics and HNUCs were categorized using the Consequences of Public Health Interventions (CONSEQUENT) framework, by level (service users, stakeholders, or system level) and degree of certainty (explicit, implicit, or potential). We followed the Joanna Briggs Institute manual and the PRISMA Reporting Guidelines for Scoping Reviews. **Results:** Of 2097 records identified, 18 primary studies met the inclusion criteria. In addition, 87 studies, identified through 35 included reviews, were analyzed. A total of 776 unique HNUCs were identified across the CONSEQUENT framework’s domains. Most were related to the domains of “Health System”, “Health” and “Acceptability and Adherence”, and predominantly affected SP participants. **Conclusions:** SP may be associated with potential HNUCs affecting users, stakeholders, and systems. Identifying and addressing these risks is essential for designing, implementing, and evaluating SP programs, and guiding future research to mitigate potential HNUCs. Our findings underscore the importance of policymakers and practitioners incorporating the routine monitoring of unintended consequences and using this evidence to inform program refinement, resource allocation, and harm mitigation strategies.

## 1. Introduction

Social prescribing (SP) emerged as an approach to support primary care in addressing patients’ non-medical social needs [1,2,3]. Broadly, SP aims to connect individuals experiencing health-related non-medical needs with appropriate community resources and services, such as social activities, volunteering opportunities, and debt advice [4,5]. The pathway typically begins with an “identifier”, often a general practitioner (GP) or other healthcare professional, who identifies non-medical needs [4,6] and initiates a referral to a “link worker”, also referred to as “connector”, “facilitator”, “well-being coordinator”, “well-being coach”, or “social prescriber” [4,5,7,8]. The link worker engages with the individual, hereinafter referred to as the service user, to explore their needs, preferences and collaboratively identify suitable support options, facilitating access to community-based services [4,9]. The process results in the development of a co-produced, non-pharmacological social prescription that links individuals to appropriate non-medical support [4,5,9,10]. Examples include financial or legal advice, exercise and lifestyle programs, and group activities, such as arts, culture, or nature-based activities [5].

Primary care is generally better equipped for biomedical concerns than social needs [11,12], partly due to limited training, time, and financial incentives to systematically assess and manage social determinants of health (SDH) [1,12,13]. Non-medical concerns are estimated to account for around 20% of GP consultation time in the United Kingdom (UK) [1]. While patients trust their GPs, this can place additional strain on primary care [1]. SDH, including social, political, and economic factors, remains a major source of health inequities [9,14]. SP has been piloted and implemented across various countries and settings [15], and has been proposed as a means of strengthening health and well-being while alleviating pressures on healthcare systems through dedicated support for non-medical needs [2,4,5,9]. SP schemes have been shown to improve well-being by reducing anxiety, loneliness, and depression [16,17,18], while also enhancing community participation, self-management, and self-efficacy [16,17,18]. These findings are particularly relevant given the growing demand for support with social issues such as loneliness and financial hardship [15,19].

Connecting sectors is crucial, as social and community services are often well positioned to address non-medical needs, but often operate separately from medical services [20]. For patients, available support may be difficult to identify or navigate, and GPs face similar challenges when seeking to connect individuals to such services [21].

Moreover, considerable heterogeneity exists in pathways, levels of support, and implementation contexts [15,22,23,24]. This heterogeneity and differences in terminology, outcome measures and the lack of a robust evidence base further complicate the identification and evaluation of harms and unintended consequences because interventions labelled as “social prescribing” may differ substantially in content, intensity, and delivery, while outcomes are inconsistently defined and measured [23,24,25,26].

Critical views raised concerns about the suitability of SP as a strategy for addressing SDH [24,27,28]. Concerns include the risk that SP may reinforce or exacerbate health inequalities by shifting responsibility onto individuals while neglecting underlying structural determinants and underinvestment in public services [24,27,28]. Potential harms to participants have also been identified, including unmet expectations regarding access to medical treatment, overdependence on services, or negative experiences within groups [24,28,29]. Non-specialist services may be unable to support individuals with complex needs [28,29]. Despite these concerns, research examining the potential harms and negative or unintended consequences (HNUCs) of SP remains limited. This research gap hinders a comprehensive assessment of the overall health impact of SP.

Unlike pharmaceutical trials, evaluations of psychosocial interventions in public health rarely investigate HNUCs and their underlying mechanisms [30,31,32]. Logic models and study designs often emphasize effectiveness and benefits, while adverse outcomes are underreported, contributing to publication bias [30,32,33]. Furthermore, the heterogeneity of SP programs and their diverse aims complicate evidence synthesis, making systematic reviews and meta-analyses particularly challenging in this context [33]. A recent umbrella review reported the potential harms of SP using the Typology of Harms Framework [34]. However, no evidence synthesis has yet systematically mapped potential HNUCs using a system-oriented framework capturing also the mechanisms and pathways through which these effects arise. A systematic focus on HNUCs is therefore essential to support safer, more effective, and better-informed policy and practice in SP.

Therefore, the objective of this review is to systematically identify and synthesize information on potential HNUCs of social prescribing interventions within the health- and social care setting, considering impacts on service users, other people involved, and the broader systems, as well as the underlying mechanisms through which these consequences may occur.

## 2. Methods

### 2.1. Applied Methodology

We conducted a scoping review to map the existing evidence following the methodological guidelines of the Joanna Briggs Institute (JBI) and PRISMA Reporting Guidelines for Scoping Reviews (ScR) [35,36]. Scoping reviews are a form of evidence synthesis particularly suited to examining the breadth and nature of evidence within a given field, including areas that have received limited research attention, such as novel interventions [35].

### 2.2. Definition of Key Terms

#### 2.2.1. Social Prescribing

Social Prescribing (SP) was defined in accordance with the operational definition by Muhl et al. [4]. SP interventions consist of at least two components: firstly, an identifier of non-medical social needs, and secondly, the creation of a social prescription together with the service user. The involvement of a link worker in the latter stage is considered an optional component. In addition, for the purpose of this review, the identification of non-medical social needs was required to occur within a healthcare setting such as general practice, primary care, pharmacies, or through community health workers.

#### 2.2.2. Harms, and Negative or Unintended Consequences

Harms and negative or unintended consequences (HNUCs) were defined as all undesirable events and adverse outcomes associated with the implementation of SP programs [33]. These encompass a broad range of potential risks, side effects, negative consequences, adverse effects, adverse reactions, and other unintended consequences affecting individuals, stakeholders, and wider systems (e.g., healthcare and social care services) [33,37].

#### 2.2.3. CONSEQUENT Framework

We used the Consequences of Public Health Interventions (CONSEQUENT) framework [32] to map the identified HNUCs. The framework is designed to support evidence-based decision-making in the design and evaluation of public health interventions by facilitating the identification and assessment of potential harms and unintended consequences. A detailed description of the CONSEQUENT framework including examples can be found in Stratil et al. [32]. Briefly, it facilitates the assessment of potential HNUCs of public health interventions across multiple levels, including health, societal and ecological outcomes. The framework consists of a consequence and a mechanism component. The consequence component includes eight main categories of potential HNUCs and 23 related sub-categories: Health, Health System, Human and Fundamental Rights, Acceptability and Adherence, Equity and Equality-Related, Social and Institutional, Economic and Resource-Related, and Ecological [32]. The mechanisms component is used to identify and describe the origins and processes through which HNUCs arise. The eight mechanisms are as follows: Through Bio-Physiological Mechanisms, (Re-)Action and Behavior Change, Perception, Experience and Assessment, Available Opportunities for (Re-)Action, Environments and Environmental Exposure, Social Norms and Practices, Economic and Market Mechanisms, and the Functioning of Systems and System Components [32]. We added an additional mechanism “Unclear or Not Reported” to the original category system of the CONSEQUENT framework to allow the systematic inclusion of HNUCs for which the underlying mechanisms were insufficiently described. Table S1 in the Supplementary Materials provides an overview of all categories and sub-categories included in the CONSEQUENT framework.

### 2.3. Literature Retrieval and Search Strategy

The selection of literature followed a two-step approach, consisting of a systematic database search and a structured reference search.

#### 2.3.1. Database Searches

A comprehensive literature search was conducted in September 2024 in EMBASE(Ovid), MEDLINE(Ovid), and APA PsycInfo to identify studies reporting HNUCs associated with SP. The database search strategy was developed for EMBASE(Ovid) by an experienced researcher (JaS) and subsequently peer reviewed by the research team using the PRESS checklist [38]. The strategy was then adapted for the remaining databases. Detailed search strategies for all databases are provided in Tables S2.1–S2.3 in the Supplementary Materials.

#### 2.3.2. Reference Searches

A structured reference search was conducted in the reviews included after the database search to identify further relevant primary studies. A full-text search of all studies on the effectiveness of SP would have been necessary but was not feasible. As an alternative, relevant primary studies, reports, and non-empirical studies were identified from systematic reviews on the effectiveness of SP. Corresponding full texts were assessed for eligibility. This approach has previously been successfully applied in other reviews on adverse and unintended consequences [39].

For one review, the authors were contacted to obtain references to sections concerning HNUCs.

### 2.4. Eligibility Criteria

We applied the following inclusion criteria: (1) The respective intervention focuses on SP within a health- or social care setting (either explicitly naming it as such or as per the operational definition of Muhl et al. [4]). (2) The study reports any type of potential harm and negative or unintended consequence of the intervention. (3) The participants receiving SP had to be older than 18 years. A detailed list of inclusion and exclusion criteria based on the PICO scheme, as we focused on a specific intervention (social prescribing), along with a hierarchy of exclusion reasons, is provided in Table S3 in the Supplementary Materials.

During title/abstract screening of the database search results, studies were excluded if they did not indicate SP, if they violated other inclusion criteria, or if they were not published in English or German. During full-text screening, studies were excluded if they were not available as full text, did not focus on SP interventions or did not report HNUCs. Studies obtained from the reference search were excluded if they did not meet the inclusion criteria.

### 2.5. Screening Process

#### 2.5.1. Selection of Studies

Screening was performed by two independent researchers (JoS, VP) using the software Rayyan (1.4.3) [40]. Disagreements were discussed and in case of disagreement resolved by a third reviewer (SP). Included primary studies proceeded to the data extraction stage. Reviews were assessed for reporting potential HNUCs to identify further relevant studies. In this structured reference search, identified studies underwent the same screening process as the initially included studies.

#### 2.5.2. Data Extraction Process

Data extraction was performed using a predefined extraction table in Microsoft Excel [41] by one researcher (JoS) and validated by a second researcher (VP).

The extracted information included authors, year of publication, title, study aim, setting, study design, intervention characteristics, sample characteristics, sample size, outcome measures, HNUCs, and reported study limitations. The extraction table can be found in Table S4 in the Supplementary Materials. HNUCs and underlying mechanisms were categorized according to the CONSEQUENT framework which also guided the interpretation of results [32]. Coding decisions were based on the CONSEQUENT framework definitions and were subject to internal verification through team discussion and consensus-based resolution of uncertainties. If the same HNUC was reported multiple times without providing additional information, it was recorded only once. An additional categorization classified each HNUC as either explicitly reported (actually occurred and clearly identified as a negative or unintended consequence), implicitly reported (described outcomes consistent with a HNUC without being labelled as such), or reported only as a potential HNUC (reported as a potential rather than observed consequence). Another category system differentiated between the occurrence of the various HNUCs as occurring either at the service user, link worker, or system level. “System” refers to healthcare, social care or other systems, including their components such as GPs, community organizations, and other stakeholders involved in the intervention. Volunteers were classified as service users as they are not formal providers. The frequency of extracted HNUCs was analyzed to map their distribution across CONSEQUENT categories.

### 2.6. Protocol and Ethical Approval

A protocol for this Scoping Review was registered on the Open Science Framework (https://osf.io/k9uej/ (accessed on 16 September 2024)). Ethical approval was obtained through the Research Committee for Scientific Ethical Questions (RCSEQ) at UMIT TIROL (Registration number 3403 on 3 April 2024).

## 3. Results

### 3.1. Search Results

**Figure 1:**
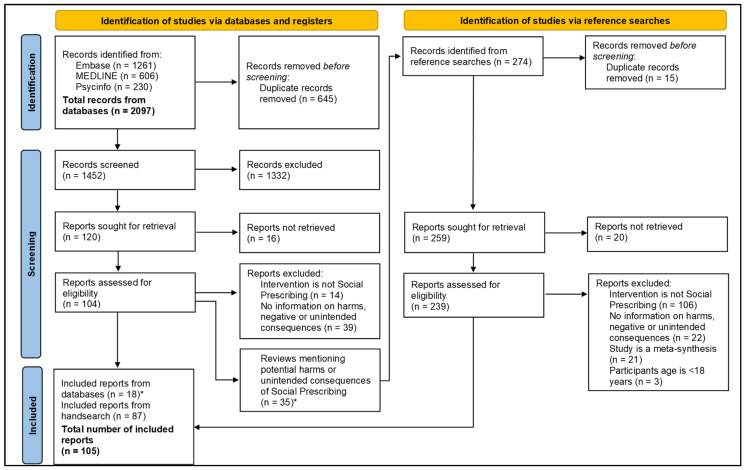
Adapted PRISMA 2020 Flow Diagram for Systematic Reviews which included Searches of Databases, Registers and Other Sources [42]. * because of their methodology, two reports were included both as primary studies identified via databases and as reviews used to identify additional primary studies in the structured hand search.

The literature search across the three databases yielded a total of 2097 records. After removing duplicates and title/abstract screening, 104 studies (including 35 reviews eligible for reference search) were included in the full-text screening. Of these studies, 14 were excluded because the intervention was not SP, and 39 studies did not provide any information on potential HNUCs. Two studies were classified as both a review and a primary study and were included as primary studies but were also used in the structured reference search. After full-text screening, 18 primary studies were included. Based on the reference search of the 35 reviews reporting or indicating potential HNUCs of SP interventions, 239 additional studies were considered for full-text screening. Of these, 87 additional studies met the inclusion criteria and were included in the scoping review. Thus, a total of 105 primary studies finally met the inclusion criteria. A detailed overview of the screening process can be found in Figure 1.

### 3.2. Characteristics of Included Studies

Most included studies were based in the UK (n = 95) [7,8,18,27,43,44,45,46,47,48,49,50,51,52,53,54,55,56,57,58,59,60,61,62,63,64,65,66,67,68,69,70,71,72,73,74,75,76,77,78,79,80,81,82,83,84,85,86,87,88,89,90,91,92,93,94,95,96,97,98,99,100,101,102,103,104,105,106,107,108,109,110,111,112,113,114,115,116,117,118,119,120,121,122,123,124,125,126,127,128,129,130,131,132,133]. Additional studies were conducted in the United States of America (n = 3) [134,135,136], the Netherlands (n = 2) [137,138], Australia (n = 1) [139], South Africa (n = 1) [140], Spain (n = 1) [141], and New Zealand (n = 1) [142]. One study provided an overview on the implementation of SP across twelve different countries [6]. Studies were published between 2000 and 2024. The majority of studies were published within the last ten years (n = 83) [6,7,18,27,43,44,45,46,47,48,49,50,53,55,56,57,59,60,61,63,64,65,67,68,69,70,71,73,74,75,76,78,79,80,81,82,84,85,86,87,88,89,90,91,92,94,95,96,97,98,99,100,101,103,104,105,106,107,108,109,110,111,112,113,115,117,118,119,121,122,124,126,127,128,130,133,134,135,136,137,139,141,142]. Most of the included studies employed a qualitative research design (n = 52) [7,44,45,47,51,52,55,59,61,63,66,67,68,70,73,75,78,79,80,81,82,87,90,94,95,96,97,98,100,101,104,105,107,108,109,110,112,113,114,115,117,119,124,126,127,128,131,132,133,137,140,141], followed by a mixed-methods design (n = 34) [18,46,48,49,50,55,56,57,58,65,69,71,72,74,84,85,86,88,91,92,93,99,102,103,111,120,121,122,123,125,129,136,138,139]. In total, 17 studies used a quantitative approach [6,53,54,60,62,64,76,77,83,89,106,116,118,130,134,135,142] and two were comments not reporting empirical data [8,27]. Of the studies included, 22 were evaluation reports [43,49,50,56,57,58,59,60,65,71,74,84,85,92,93,99,103,111,120,122,125,129] and two were part of a doctoral thesis [119,136].

Studies covered a variety of settings, with most studies and participants in primary care (n = 42) [7,45,46,50,52,56,57,63,64,65,66,70,71,72,75,77,79,80,81,85,88,89,90,91,92,93,99,103,105,106,107,114,120,122,124,125,126,135,137,139,141,142], the third sector (community and voluntary organizations, as part of SP programs) (n = 31) [51,59,60,62,69,73,76,78,82,83,84,86,94,96,97,98,102,112,113,116,117,118,119,121,123,127,128,129,131,132,138], or a combination of both settings (n = 13) [46,47,48,49,55,58,74,101,109,110,115,133,136]. In total, 15 studies used information gathered among stakeholders (e.g., from funding bodies or link workers) without specifying a clear pathway of SP delivery [6,18,27,43,44,53,61,67,68,87,95,100,104,111,130]. A smaller portion of studies was located at emergency departments or clinics (n = 3) [54,134,140], and one study [108] involved pharmacists as a potential access point to SP. A more detailed overview of the characteristics of included studies is provided in Table S5. A full list of excluded studies at the full-text screening stage with their exclusion reasons is provided in Table S6, and the PRISMA-ScR checklist in Table S7 in the Supplementary Materials.

### 3.3. Identified Harms, and Negative or Unintended Consequences Across Studies

**Figure 2:**
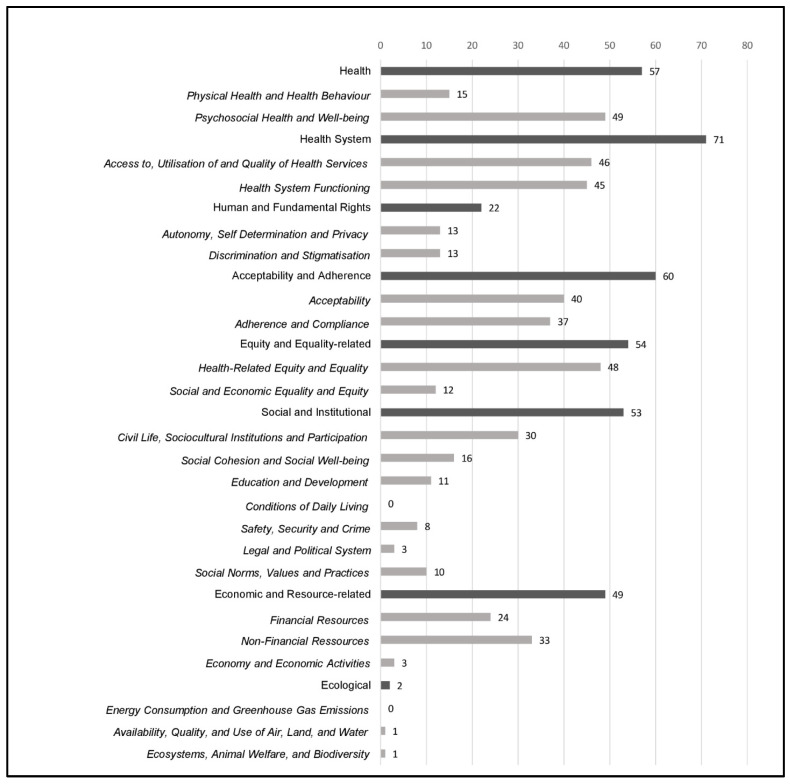
Number of Studies Reporting Harms, or Negative or Unintended Consequences across the Categories and Sub-categories of the CONSEQUENT Framework [32]. Dark bars show the number of studies reporting on harms or negative and unintended consequences within each main category of the CONSEQUENT framework; bright bars show the number reporting on respective sub-categories (in italics). Totals for main categories may differ from the sum of sub-categories, as studies may only report on certain sub-categories.

Within the 105 studies included, 776 passages referring to actual or potential HNUCs were identified. HNUCs were found for all eight main categories of the CONSEQUENT framework. The most frequent categories were “Health System”, “Acceptability and Adherence”, and “Equity and Equality-Related”, identified in 71, 60, respectively, and 54 of the included studies. Except for two sub-categories (Conditions of Daily Living, Energy Consumption and Greenhouse Gas Emissions), we were able to allocate potential HNUCs of SP interventions to all sub-categories of the framework. Furthermore, all mechanisms of the framework were represented. Figure 2 shows the number of studies reporting HNUCs for each main category (dark bars) and for the respective sub-categories (bright bars) below.

### 3.4. An Overview of the Main Categories of the CONSEQUENT Framework

**Figure 3:**
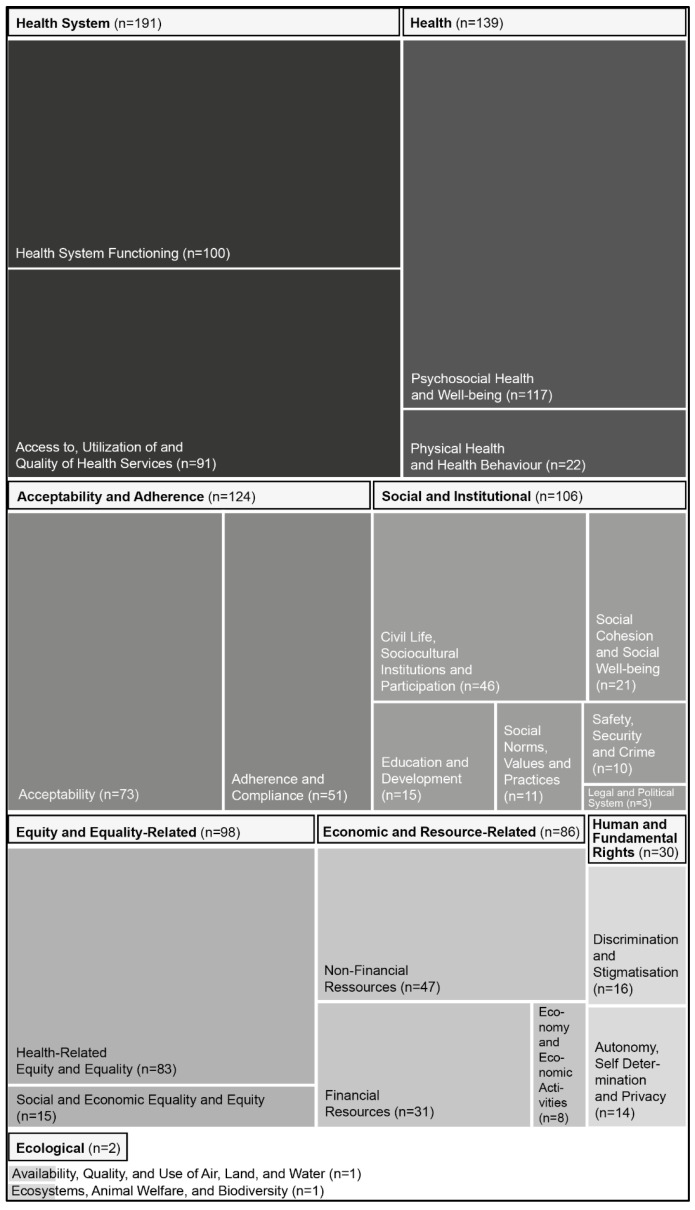
Tree Map—Overview about all Identified HNUCs across the Categories and Sub-categories of the CONSEQUENT Framework [32]. Each tile represents a sub-category of the CONSEQUENT framework for which HNUCs were identified. Tile size corresponds to their proportion relative to all identified HNUCs, while the numeric value refers to the total number of HNUCs identified within that category or sub-category. The sub-category of Conditions of Daily Living within Social and Institutional and the sub-category of Energy Consumption and Greenhouse-Gas Emission did not contain any HNUCs and are therefore not represented in this chart.

To unpack the nuances behind the categories of the framework, the following sections examine how HNUCs appeared across contexts and stakeholder groups. Among all 776 identified HNUCs, the categories most frequently used to cluster the identified HNUCs were “Health System”, with 191 identified HNUCs (25% of all HNUCs); 139 HNUCs (18%) were related to “Health”, and 124 (16%) were related to “Acceptability and Adherence”. Together, these three categories account for 58% of all HNUCs. The most frequently used mechanisms were “Through the Functioning of Systems and System Components” (n = 187; 24%), “Through Available Opportunities for (Re-)Action” (n = 170; 22%) and “Through Perception, Experience and Assessment” (n = 109; 14%). The fewest HNUCs were found within the category of “Ecological” (n = 2; 0.3%) followed by the category of “Human and Fundamental Rights” (n = 30; 4%). The tree map shown in Figure 3 provides an overview of the various categories and sub-categories. Each tile represents a sub-category of the CONSEQUENT framework for which HNUCs were identified. Tile size corresponds to its proportion relative to all identified HNUCs, while the numeric value refers to the total number of HNUCs identified within that category or sub-category.

### 3.5. An Overview of the Level of Occurrence of HNUCs

Most HNUCs (62%) were related to SP service users (e.g., [99,104,133]). Service users were classified as individuals receiving SP, including those individuals volunteering in roles, such as befriending schemes or peer-support (e.g., [65,96]). HNUCs affecting link workers accounted for 14% of all HNUCs (e.g., [7,47,140]), while 25% of all HNUCs affected the overall system (e.g., [48,61,64]).

Regarding reporting type, 43% were explicitly reported during SP implementation (e.g., [44,54,134]). These included, for example, psychological distress among service users or link workers while participating in the intervention (e.g., [51,70,86,123]). Implicitly reported HNUCs account for 32%, reflecting mentioned negative impacts that were speculative or not explicitly referred to the actual HNUC. These included indications of reduced benefit or accessibility [53,54,67,82,83,89,93,134,135]. Potential negative long-term effects were also classified as implicitly reported HNUCs, in cases where the processes leading to certain HNUCs may have already started but have not yet fully manifested. Examples include mention of a high workload or complex casework for link workers, which could negatively affect their mental health in the long run [47,74,78,79,81,82,109,125,136]. The remaining 25% of HNUCs were reported as potential and anticipated HNUCs, typically identified through stakeholder risk considerations of certain SP schemes [73,74,104,129]. These included concerns about potential negative effects following withdrawal of support at the end of SP interventions [27,58,74,96,105,138].

### 3.6. An Overview of the Sub-Categories of the CONSEQUENT Framework

The results for the five most frequent sub-categories including number of HNUCs, mechanisms, and exemplary quotes from the corresponding studies are shown in Table 1.

## 4. Discussion

### 4.1. Principal Findings

Literature frames SP as an intervention intended to achieve a broad range of public health objectives. Different models of SP delivery with various levels of intensity are being employed [22], interacting with multiple systems, such as the healthcare and social care, the community sector and the social systems of participants along the pathway [9]. Our findings demonstrate that SP is not without potential harms, and negative or unintended consequences. While most identified HNUCs directly affected service users and link workers, they also extended to other stakeholders and the broader system. This is consistent with the umbrella review of Cooper et al. (2026) [34]. The authors identified harms across psychological, group/social, equity or opportunity domains based on 16 included reviews [34]. In contrast, to the umbrella review approach, our synthesis draws on a broader evidence base including primary studies and additional sources identified through backward citation searching, enabling the mapping of mechanisms through which these consequences may arise. The findings suggest that systemic factors and interactions with existing structures are the key drivers of many HNUCs. In addition, HNUCs arise from a lack of acceptance among key stakeholders such as GPs or service users, often due to differing expectations or perceptions that SP is inadequate to address social needs [65,68,74]. Existing services may perceive SP as competition [50,137], or may be concerned about inadequate or unfunded referrals [47,48]. Concerns have also been raised about stigmatization or exclusion for service users, for example “The link workers felt some groups might exclude people based on their disability due to prejudice and concern about managing a person with a complex condition.” [112] or “Barriers to taking part in such schemes were identified including the stigma of being identified as lonely and the associated stereotypes of people who use services for loneliness or isolation, and not wishing to see themselves within this group.” [90].

However, there is a lack of systematic assessment and reporting of HNUCs within SP using quantitative measures, a finding in line with a recently published umbrella review [143]. Establishing and routinely applying standardized quantitative measures for HNUCs should be a priority for future research, as this would improve comparability across studies, support meta-analytic synthesis, and provide more robust evidence to guide policy decisions and the safe implementation of SP programs. Included studies were from seven different regions with an overrepresentation of the UK, likely reflecting its leading role in SP development and implementation [144,145]. Broader international evidence would be of relevance, as the implementation, delivery, and integration of SP vary considerably across countries according to differences in health and social care systems, resource availability, referral pathways, and cultural contexts. Such contextual factors may influence both the types and frequency of HNUCs observed, particularly those related to access, equity, service capacity, and stakeholder experiences. The growing international uptake of SP highlights the importance of examining HNUCs across diverse settings to better understand the transferability of findings and identify context-specific risks and mitigation strategies.

Due to the high proportion of HNUCs arising from systemic factors, a more international and structured evidence base using established frameworks for classifying HNUCs may help to identify consequences that have not yet been considered within specific contexts or studies and may support efforts to mitigate or prevent them.

Included studies indicate that most HNUCs relate to the functioning of healthcare systems, the psychosocial health and well-being of individuals involved in SP, and the accessibility of SP or subsequent services. The limited availability of appropriate or accessible services negatively affected service users attempting to access support, and link workers referring options. This might result from an over-utilization of certain services or SP itself [47,49,79,125,127,133], and is especially critical when it affects vulnerable groups, experiencing challenges in accessing services due to their conditions or sociodemographic characteristics [50,63,65,72,82,85,86,87,100,111,116,127]. This finding conflicts with the actual goals of SP to help people access relevant support, and with public health ethics of promoting health equity. A reciprocal relationship between HNUCs and conflicts with existing systems and services in the healthcare and community sector might occur when SP is used to fill gaps or substitute for overstretched services [124]. As a result, SP services might become overutilized or experience reduced quality, including the provision of support without the necessary resources such as qualified staff [79,82,124].

Beyond the specific HNUCs identified in the included studies, the findings raise broader questions regarding how SP is implemented across interconnected health, social care, and community systems. The identified HNUCs suggest that SP operates across sectors that are often disconnected, which may create challenges in building collaborations between systems with different values, priorities, and ethical foundations. The medical system traditionally focuses on identifying biophysiological or biomechanical causes of diseases, treating with standardized, evidence-based protocols, often including pharmaceuticals [146]. Psychosocial needs are acknowledged but often unmet, as physicians typically lack the time, incentives, and tools to address them [1,13]. Individuals may often be pathologized by viewing these needs as conditions requiring treatment, or these needs are disregarded altogether [11,12]. Conversely, the institutionalized social and community sector often lacks the capacity to meet medical needs [50,59,78], being underfunded and reliant on public funding or donations [70].

Our findings also show that SP may adversely affect the health and well-being of service users and link workers. Psychological distress among service users while taking part in the intervention (e.g., [70,123]) or link workers developing stress-related issues due to high workload or emotionally challenging cases (e.g., [74,86]) may lead to immediate or long-term consequences.

Given this, SP may result in HNUCs extending beyond the individual level, with stakeholders perceiving SP as either a beneficial innovation or a problematic disruption.

General practitioners may benefit from easier or more diverse referral pathways but need to know them, while link workers may find meaning in supporting users yet also experience stress and strain. Service users might be dissatisfied if they are expected to receive medical treatment, and community organizations may struggle to maintain service quality if demand exceeds capacity. Diverse expectations across system components can, therefore, influence overall SP outcome, highlighting the need for an approach that considers the entire system and all sub-systems. SP itself might be confronted with the need to achieve enough onward referrals as a performance indicator [86,94], risking the medicalization of social needs and the institutionalization of users [147]. Many informal resources within communities and among individuals might then be neglected, thereby undermining the main intention of SP as a person-centered and empowering approach [148,149].

Our findings align with these critiques and demonstrate that SP can only be successful if systems and stakeholders have sufficient resources and readiness to support SP implementation. To deliver personalized support, constraints across systems, communities, and individuals must be considered, and link workers’ roles must extend beyond simple signposting. This also means assessing service users’ personal capacities, individual contexts, and the resources available within communities. Otherwise, responsibility for structural factors may be shifted onto individuals [27], risking HNUCs such as poorer health or diminished trust in health care. Without a careful systemic approach, SP may risk exacerbate health inequities rather than addressing them [150].

Although this review focuses on potential HNUCs, evidence overall shows positive reception among service users and other stakeholders (e.g., [2,88,151,152]), indicating that SP can play an important role in addressing social needs via non-medical solutions and the utilization of community resources. Moreover, the challenges identified are not necessarily unique to SP. Similar negative experiences have been reported in other interventions, such as individuals undergoing psychological treatment [153] or healthcare professionals experiencing distress in challenging patient situations or due to limited resources [154,155,156]. The occurrence of negative outcomes should not be interpreted as evidence of failure but as a signal for where systems and services may require support. Our aim is not to suggest that SP necessarily produces harmful effects across all contexts, but to inform future planning and implementation. Therefore, policymakers and practitioners should consider the routine monitoring of potential HNUCs across service users, providers, and system levels, with particular attention to the categories and mechanisms identified in this review. Early identification of unintended consequences may support timely mitigation strategies, including adequate staff training, clear referral pathways, appropriate resource allocation, and the ongoing evaluation of program implementation.

### 4.2. Strengths and Limitations

One of the main strengths of this review is the systematic approach used for the literature search in bibliographic databases and the use of backward reference searches of relevant reviews. A peer-reviewed search code that included the use of validated search filters was developed and was tested against key articles in the field of SP, ensuring that important contributions were not overlooked. Screening was performed independently and blinded by two reviewers, and disagreements were discussed with a third reviewer. Data extraction was also double-checked by two independent researchers to support objectivity and reliability. The CONSEQUENT framework served as a tool to guide the screening process of HNUCs in various categories, as well as to structure the data extraction process. In addition, we aimed to enhance quality and transparency by registering the review with the Open Science Framework and aligning with internationally recognized standards such as the JBI guidelines for evidence synthesis and the PRISMA-ScR checklist.

As with all scoping reviews, our study has several limitations. The search was restricted to publications in English and German and only English search terms. Relevant studies published in other languages and indexed in additional interdisciplinary databases might have been overlooked. In addition, reliance on backward reference searching of included reviews to identify additional studies may have led to missing less cited or non-indexed studies. As we did not conduct a structured search of the grey literature, implementation reports, policy documents, and other practice-based evidence may also have been missed, which could have introduced publication bias or resulted in an incomplete representation of HNUCs. The inclusion of two commentaries represented a deviation from the original eligibility criteria. As these publications were not identified through a dedicated search strategy, other relevant commentaries may have been overlooked. As the literature searches were conducted in September 2024, studies published thereafter were not included and more recent evidence may therefore not be reflected in this review. However, we did not observe a general pattern within the data, that would indicate that there would be a systematic deviation between the more recent and earlier publications, indicating that the results would have changed following such an update. Given the large volume and heterogeneity of the included studies, a detailed thematic synthesis was not feasible; therefore, the evidence was systematically mapped and summarized according to the predefined HNUC framework. In line with scoping review methodology, no formal critical appraisal of included studies was undertaken, as the aim was to map the breadth of evidence rather than assess study quality. In addition, the coding and categorizing of HNUCs is inherently subjective. Although calibration exercises were employed to harmonize classification decisions among reviewers, some variation in interpretation remained. Furthermore, HNUCs were coded by a single reviewer and verified by a second reviewer, rather than being independently coded in duplicate. While independent double coding is generally regarded as the methodological gold standard and may have reduced the risk of confirmation bias, the chosen approach reflected a pragmatic decision given the substantial resources required for full double coding. The use of more qualitative data analysis or more advanced coding frameworks, such as the “best fit” framework [157] or thematic synthesis [158], might have enhanced reproducibility and further reduced subjectivity.

### 4.3. Suggestions for Future Research

Although inherent to the scoping review methodology and its scope, this review does not allow for conclusions about causality or the magnitude of specific harms and negative or unintended consequences. Rather, it provides an overview of the areas where such effects may occur. Importantly, the frequencies presented reflect the frequency of reported of HNUCs within the included studies, rather than their actual prevalence or incidence in SP interventions. Future research could benefit from employing mixed-methods synthesis approaches with a focus on selected, standardized outcome measures to better quantify the prevalence and severity of specific HNUCs. However, achieving this depends on building a more robust evidence base supported by high-quality data and the consistent application of validated outcome measures specific to these harms. Developing standardized reporting of HNUCs and improving data quality will be essential steps toward drawing directed conclusions and informing effective strategies to mitigate these HNUCs.

## 5. Conclusions

We identified a broad range of HNUCs associated with SP within the healthcare setting across all eight main categories of the CONSEQUENT framework and most of its subcategories. Most frequently, HNUCs affected the functioning of the healthcare system, followed by direct health-related effects for service users and providers. Our findings demonstrate that SP entails potential risks and requires careful consideration at multiple levels and requires careful consideration in both policy and practice. Policymakers, clinicians, healthcare workers, and other professionals involved in SP should carefully weigh these potential HNUCs and expected benefits against potential risks or harms, including assessing resource availability at system and individual level and ensuring adequate allocation where SP is implemented. The transparent reporting of benefits and HNUCs in SP will contribute to the evidence base, allows for service adaptation and can support potential users by informing them about risks and support decision-making regarding the appropriate indication of SP or whether alternative interventions may be more appropriate. Future research should prioritize the development of standardized methods for identifying, measuring, and reporting HNUCs in SP to better understand their prevalence, severity, and mechanisms. In parallel, the implementation of SP programs should incorporate the routine monitoring of unintended consequences to support safer design, more efficient resource allocation, and continuous service improvement.

## Figures and Tables

**Table 1:** Results for the five most frequent sub-categories of the CONSEQUENT framework [32].

Main Category: Sub-Category	No. of HNUCs (% of All)	No. of HNUCs Affecting Stakeholders	Mechanism (Top Three)	Exemplary Quotes from Studies
Health: Psychosocial Health and Well-being	117 (15%) (70 explicit, 12 implicit, 35 potential)	79 service users, 35 link workers, 3 system levels	- Perception, Experience and Assessment, - Environments and Environmental Exposure - Functioning of Systems and System Components	“Before, during, and after this interaction Eddie’s anxiety and shame was evident. For instance, in the meeting he apologised profusely for forgetting his bank statements and chastised himself for being stupid. Afterwards he said he felt ‘ashamed and embarrassed’.” (111: p. 5)
Health System: Health System Functioning	100 (13%) (41 explicitly, 38 implicitly, 21 potential)	16 service users, 31 link workers, 53 system levels	- Functioning of Systems and System Components - Available Opportunities for (Re-)Action - Economic and Market Mechanisms	“The mis-match between supply and demand may have contributed to low uptake of some funded services and, in some cases, conflict within the system. With, for example, new social prescribing offers duplicating and undermining established Occupational Therapy provision.” (104: p. 128)
Health System: Access to, Utilization of and Quality of Health Services	91 (12%) (44 implicitly, 33 explicitly, 14 potential)	68 service users, 8 link workers, 15 system levels	- Available Opportunities for (Re-)Action - Through the Functioning of Systems and System Components - Perception, Experience and Assessment	“Some respondents felt that the involvement of health professionals in the referral process was unnecessary, feeling that they ultimately served as gatekeepers, rationing access to prevent the schemes from becoming overwhelmed, rather than actively promoting the service.” (137: p. 751)
Equity and equality-related: Health related equity and equality	83 (11%) (31 explicit, 32 implicit, 20 potential)	71 service users, 1 link workers, 11 system levels	- Available Opportunities for (Re-)Action, - Unclear or Not Reported, - Functioning of Systems and System Components	“The ubiquity of online delivery has multiple implications, including creating greater social and health divisions between the digitally privileged and disadvantaged.” [68]
Acceptability and adherence: Acceptability	73 (9%) (30 explicit, 32 implicit, 11 potential)	50 service users, 23 system levels	- Perception, Experience and Assessment, - Social Norms and Practices, - Functioning of Systems and System Components	“Like all social prescribing offers, there is some evidence that the public do not consider it to be a legitimate medical referral. This may be especially the case in the context of chronic underfunding of mental health services, with a non-medical referral perceived to be a ‘cop-out’ and failure to provide more accepted treatment options. This is likely to affect both uptake of referral but also the effectiveness of the programme itself.” [74]

## Data Availability

No new data were created or analyzed in this study. Data sharing is not applicable to this article.

## Supplementary Materials

The following supporting information can be downloaded at: https://www.mdpi.com/article/10.3390/healthcare14131947/s1, Table S1: Categories and sub-categories of HNUCs within the CONSEQUENT Framework [1]; Table S2.1: Search Strategy used in EMBASE(Ovid); Table S2.2: Search Strategy used in MEDLINE(Ovid); Table S2.3: Search Strategy used in APA PsycInfo; Table S3: Detailed inclusion and exclusion criteria/ Study selection process; Table S4: Extraction Table; Table S5: Characteristics of Included Studies; Table S6: List of Studies Excluded at the Full-Text Screening Stage from the Database Search; Table S7: Preferred Reporting Items for Systematic reviews and Meta-Analyses extension for Scoping Reviews (PRISMA-ScR) Checklist [106,159].
