# Supplementary material for: Harms and Negative or Unintended Consequences of Social Prescribing: A Scoping Review
Source: Healthcare (Basel). 2026 Jul 1;14(13):1947. doi: 10.3390/healthcare14131947 (PMC13362470; doi:10.3390/healthcare14131947)
Supplement: Supplementary file 1 [file healthcare-14-01947-s001.zip › healthcare-4333587-supplementary.pdf]

Appendix

Table S1: Categories and sub-categories of HNUCs within the CONSEQUENT Framework [1]

No.	Main category	No.	Sub-category
1	Health	1.1	Physical Health and Health Behavior
		1.2	Psychosocial Health and Well-being
2	Health System	2.1	Access to, Utilization of and Quality of Health Services
		2.2	Health System Functioning
3	Human and Fundamental Rights	3.1	Autonomy, Self-determination and Privacy
		3.2	Discrimination and Stigmatization
4	Acceptability and Adherence	4.1	Acceptability
		4.2	Adherence and Compliance
5	Equity and Equality-Related	5.1	Health-Related Equity and Equality
		5.2	Social and Economic Equality and Equity
6	Social and Institutional	6.1	Civil Life, Sociocultural Institutions and Participation
		6.2	Social Cohesion and Social Well-being
		6.3	Education and Development
		6.4	Conditions of Daily Living
		6.5	Safety, Security and Crime
		6.6	Legal and Political System
		6.7	Social Norms, Values and Practices
7	Economic and Resource-Related	7.1	Financial Resources
		7.2	Non-Financial Resources

		7.3	Economy and Economic Activities
8	Ecological	8.1	Energy Consumption and Greenhouse Gas Emissions
		8.2	Availability, Quality, and Use of Air, Land, and Water
		8.3	Ecosystems, Animal Welfare, and Biodiversity

Table S2.1: Search Strategy used in EMBASE(Ovid)

#	Searches	Results
1	(social adj3 (prescrib* or referr*)).ti,ab,kw.	2398
2	((nature* or outdoor* or out-door*) adj2 (prescrib* or referr*)).ti,ab,kw.	260
3	(communit* adj2 (prescrib* or referr*)).ti,ab,kw.	3444
4	(non adj1 (medical or pharma* or therpeutic* or drug* or clinical*) adj2 (prescrib* or referr*)).ti,ab,kw.	615
5	((nonmedical or nonpharma* or nontherpeutic* or nondrug* or nonclinic*) adj2 (prescrib* or referr*)).ti,ab,kw.	125
6	1 or 2 or 3 or 4 or 5	6687
7	meta analysis/	328456
8	Meta-Analysis as Topic/	43981
9	exp Review Literature as Topic/	271473
10	((meta adj analy$) or (meta adj synth$) or metaanalys$ or metasynth$).tw.	402461
11	((evidence adj2 synth$) or evidencesynth$).tw.	18721
12	((systematic or scoping or umbrella) adj2 (review$1 or overview$1)).tw.	458167
13	(cochrane or embase or (psychlit or psyclit) or (psychinfo or psycinfo) or (cinahl or cinhal) or science citation index or bids or cancerlit).ab.	338975
14	(reference list$ or bibliograph$ or hand-search$ or relevant journals or manual search$).ab.	71883
15	(selection criteria or data extraction).ab.	90796
16	Review/	3058709
17	7 or 8 or 9 or 10 or 11 or 12 or 13 or 14 or 15 or 16	3826448
18	6 and 17	620
19	(complication* or Injuring or injured).ti,ab,kw.	1914918
20	((risk* or rate*) adj4 (injur* or fall*)).ti,ab,kw.	107007
21	((symptom* or condition* or health* or stress* or marginali* or equit* or equal*) adj5 (negative* or worse* or deteriorat* or declin* or	414577

	degrad* or Exacerbat* or degenerat* or aggravat* or decay* or impair* or regress* or erode*)).ti,ab,kw.	
22	adverse outcome/	73320
23	adverse event/ or exp adverse device effect/	276790
24	patient harm/	3246
25	(sideeffect* or side effect*).ti,ab,kw.	475101
26	maleficence*.ti,ab,kw.	1001
27	((harm* or adverse or unintended or unintentional or Unanticipated or unwanted or paradoxical or iatrogenic) adj2 (effect or effects or reaction* or event* or outcome* consequence*)).ti,ab,kw.	924139
28	((non or "not") adj1 (intended or intentional or anticipat*) adj2 (effect or effects or reaction* or event* or outcome* consequence*)).ti,ab,kw.	63
29	19 or 20 or 21 or 22 or 23 or 24 or 25 or 26 or 27 or 28	3759749
30	6 and 29	716
31	18 or 30	1261

Table S2.2: Search Strategy used in MEDLINE(Ovid)

#	Searches	Results
1	(social adj3 (prescrib* or referr*)).ti,ab,kw.	1606
2	((nature* or outdoor* or out-door*) adj2 (prescrib* or referr*)).ti,ab,kw.	151
3	(communit* adj2 (prescrib* or referr*)).ti,ab,kw.	2127
4	(non adj1 (medical or pharma* or therpeutic* or drug* or clinical*) adj2 (prescrib* or referr*)).ti,ab,kw.	304
5	((nonmedical or nonpharma* or nontherpeutic* or nondrug* or nonclinic*) adj2 (prescrib* or referr*)).ti,ab,kw.	80

6	1 or 2 or 3 or 4 or 5	4170
7	(systematic review or meta-analysis).pt.	359597
8	Meta-Analysis as Topic/	24554
9	meta-analysis/ or systematic review/ or systematic reviews as topic/ or meta-analysis as topic/ or "meta analysis (topic)"/ or "systematic review (topic)"/ or exp technology assessment, biomedical/ or network meta-analysis/	401921
10	((meta adj analy$) or (meta adj synth$) or metaanalys$ or metasynth$).tw.	318029
11	((evidence adj2 synth$) or evidencesynth$).tw.	16639
12	((systematic or scoping or umbrella) adj2 (review$1 or overview$1)).tw.	381815
13	(cochrane or embase or (psychlit or psyclit) or (psychinfo or psycinfo) or (cinahl or cinhal) or science citation index or bids or cancerlit).ab.	285037
14	(reference list$ or bibliograph$ or hand-search$ or relevant journals or manual search$).ab.	58358
15	(selection criteria or data extraction).ab.	73323
16	Review/	3374527
17	7 or 9 or 10 or 11 or 12 or 13 or 14 or 15	672171
18	6 and 17	259
19	(complication* or Injuring or injured).ti,ab,kw.	1300992
20	((risk* or rate*) adj4 (injur* or fall*)).ti,ab,kw.	80420
21	((symptom* or condition* or health* or stress* or marginali* or equit* or equal*) adj5 (negative* or worse* or deteriorat* or declin* or	299957

	degrad* or Exacerbat* or degenerat* or aggravat* or decay* or impair* or regress* or erode*)).ti,ab,kw.	
22	Adverse Outcome Pathways/ or "Drug-Related Side Effects and Adverse Reactions"/	40053
23	Long Term Adverse Effects/	808
24	patient harm/	240
25	(sideeffect* or side effect*).ti,ab,kw.	319003
26	maleficence*.ti,ab,kw.	768
27	((harm* or adverse or unintended or unintentional or Unanticipated or unwanted or paradoxical or iatrogenic) adj2 (effect or effects or reaction* or event* or outcome* consequence*)).ti,ab,kw.	592612
28	((non or "not") adj1 (intended or intentional or anticipat*) adj2 (effect or effects or reaction* or event* or outcome* consequence*)).ti,ab,kw.	50
29	19 or 20 or 21 or 22 or 23 or 24 or 25 or 26 or 27 or 28	2450133
30	6 and 29	378
31	18 or 30	606

Table S2.3: Search Strategy used in APA PsycInfo

#	Searches	Results
1	TI ((prescrib* OR referr*) N3 (social)) OR AB ((prescrib* OR referr*) N3 (social))	1792
2	TI ((nature* OR outdoor* OR out-door*) N2 (prescrib* OR referr*)) OR AB ((nature* OR outdoor* OR out-door*) N2 (prescrib* OR referr*))	168

3	TI ((prescrib* OR referr*) N2 (communit*)) OR AB ((prescrib* OR referr*) N2 (communit*))	1391
4	TI ((non) N1 (medical OR pharma* OR therapeutic* OR drug* OR clinical*) N2 (prescrib* OR referr*)) OR AB ((non) N1 (medical OR pharma* OR therapeutic* OR drug* OR clinical*) N2 (prescrib* OR referr*))	196
5	TI ((nonmedical OR nonpharma* OR nontherapeutic* OR nondrug* OR nonclinic*) N2 (prescrib* OR referr*)) OR AB ((nonmedical OR nonpharma* OR nontherapeutic* OR nondrug* OR nonclinic*) N2 (prescrib* OR referr*))	66
6	S1 OR S2 OR S3 OR S4 OR S5	3526
7	PT (systematic review OR meta-analysis)	0
8	DE Meta Analysis	5500
9	DE meta-analysis OR DE systematic review OR TI meta analysis (topic) OR AB meta analysis (topic) OR TI systematic review (topic) OR AB systematic review (topic) OR DE technology assessment, biomedical OR DE network meta-analysis	1981
10	TI ((meta) N (analy* OR meta) N (synth* OR metaanalys* OR metasynth*)) OR AB ((meta) N (analy* OR meta) N (synth* OR metaanalys* OR metasynth*))	40
11	TI ((evidence) N2 (synth* OR evidencesynth*)) OR AB ((evidence) N2 (synth* OR evidencesynth*))	4056
12	TI ((systematic OR scoping OR umbrella) N2 (review* OR overview*)) OR AB ((systematic OR scoping OR umbrella) N2 (review* OR overview*))	67033
13	AB (cochrane OR embase OR (psychlit OR psyclit) OR (psychinfo OR psycinfo) OR (cinahl OR cinhal) OR "science citation index" OR bids OR cancerlit)	

14	TI (reference list* OR bibliograph* OR hand-search* OR relevant journals OR manual search*) OR AB (reference list* OR bibliograph* OR hand-search* OR relevant journals OR manual search*)	26197
15	TI (selection criteria OR data extraction) OR AB (selection criteria OR data extraction)	10039
16	PT Review	0
17	S7 OR S9 OR S10 OR S11 OR S12 OR S13 OR S14 OR S15	5390584
18	S6 AND S17	3520
19	TI (complication* OR injuring OR injured) OR AB (complication* OR injuring OR injured)	43464
20	TI ((risk* OR rate*) N4 (injur* OR fall*)) OR AB ((risk* OR rate*) N4 (injur* OR fall*))	13180
21	TI ((symptom* OR condition* OR health* OR stress* OR marginali* OR equit* OR equal*) N5 (negative* OR worse* OR deteriorat* OR declin* OR degrad* OR exacerb* OR degenerat* OR aggravat* OR decay* OR impair* OR regress* OR erode*)) OR AB (symptom* OR condition* OR health* OR stress* OR marginali* OR equit* OR equal*) N5 (negative* OR worse* OR deteriorat* OR declin* OR degrad* OR exacerb* OR degenerat* OR aggravat* OR decay* OR impair* OR regress* OR erode*))	129061
22	MA Adverse Outcome Pathway OR MA drug-related side effects and adverse reactions OR MA side effects OR MA adverse drug reactions OR MA adverse effects	1516

23	DE long term adverse effects	20
24	TI patient harm OR AB patient harm	2403
25	TI (sideeffect* OR side effect) OR AB (sideeffect* OR side effect)	38497
26	TI maleficence* OR AB maleficence*	277
27	TI ((harm* OR adverse OR unintended OR unintentional OR Unanticipated OR unwanted OR paradoxical OR iatrogenic) N2 (effect OR effects OR reaction* OR event* OR outcome* OR consequence*)) OR AB ((harm* OR adverse OR unintended OR unintentional OR Unanticipated OR unwanted OR paradoxical OR iatrogenic) N2 (effect OR effects OR reaction* OR event* OR outcome* OR consequence*))	70264
28	TI ((non OR not) N1 (intended OR intentional OR anticipat*) N2 (effect OR effects OR reaction* OR event* OR outcome* OR consequence*)) OR AB ((non OR not) N1 (intended OR intentional OR anticipat*) N2 (effect OR effects OR reaction* OR event* OR outcome* OR consequence*))	166
29	S19 OR S20 OR S21 OR S22 OR S23 OR S24 OR S25 OR S26 OR S27 OR S28	279434
30	S6 AND S29	230
31	S18 OR S30	230

Table S3: Detailed inclusion and exclusion criteria/ Study selection process

	Inclusion criteria	Exclusion criteria
**Population**	Adults, participants > 18 years	Children, participants < 18 years
**Intervention**	• Social prescribing in health- or social care<br>• Prescribed by any provider, e.g., physicians/health professionals or other organizational representatives, irrespectively of the treatment goal.<br>• No specific setting.	Other interventions than social prescribing.
**Comparison**	No intervention	-
**Outcome**	Harms, negative consequences, adverse events, adverse or other unintended consequences	Studies not reporting any harms, negative consequences, adverse events, adverse or other unintended consequences
**Study design**	• Reviews assessing the effects of Social Prescribing interventions<br>• Primary studies: Any empirical study design	-
**Others**	• English or German language<br>• No restriction in publication year<br>• full-text available	• Not related to humans<br>• Not published in English or German language<br>• No full-text available

We used the following selection criteria:
1. Is the articles' full text available in English/German?
2. Is this a primary, full-text article or a relevant review?
3. Is the intervention within the research area?
4. Is there a potential harm, negative consequence, adverse event related to the SP activity?

Hierarchy of Exclusion Criteria:

- EXC SP
- EXC Age
- EXC harms
- EXC FT
- EXC Lang
- EXC healthcare

Table S4: Extraction Table

Table S4 shows an illustrative example of coded passages from one identified study. Categories and sub-categories of HNUCs, as well as the mechanism, are based on the CONSEQUENT Framework [1]. Mechanisms are: 1 bio-physiological mechanisms; 2 (re-)action and behaviour change; 3 perception, experience and assessment; 4 available opportunities for (re-)action; 5 environments and environmental exposure; 6 social norms and practices; 7 economic and market mechanisms; 8 the functioning of systems and system components.

Author	Publication year	Title	Study aim	Setting	Study design	Intervention characteristics	Sample Characteristics	Sample Size	Outcome measures	Limitations
**Al-Khudairy et al**	2022	Evidence and methods required to evaluate the impact for patients who use social prescribing: a rapid systematic review and qualitative interviews	This study investigates what evidence and methods would be needed to assess and understand the impact of a link worker for patients who use social prescribing in England.	England (South West, South East, North West, North East, West Midlands, East Midlands and London)	Rapid systematic review and qualitative interviews	Social prescribing (several domains including social isolation, housing and weight management)	Social prescribers/link workers, regional leads, learning coordinators and three voluntary community and social enterprise sector workers: a programme manager, a freelance director of a voluntary organisation and a manager of a service providing telephone support during the COVID-19 pandemic. In addition, views of topic experts, patient and public representatives, stakeholders and academic colleagues were captured.	25 participants	Semi-structured interviews	This was a rapid systematic review that did not include a systematic quality assessment of studies. COVID-19 had an impact on the shape of the service. We were not able to examine the potential causal mechanisms in any detail.

Includes Information about harms, negative consequences, adverse events (Evidence Grade: 1= explicitly; 2=implicitly included; 3=reported as potential effect)	Level (LW=Linkworker; SU=Service User; SYS=System)	Quote	Main category	Sub-category	Mechanism
1	SYS	"Heterogeneous service delivery: Some organisations have been delivering SP before adaptation by the NHS, which is perceived as destabilising the previous delivery systems." p. 82	2	2.2	8
2	SYS	"large differences in terms of available onward referral services and engagement with the PCN" p. 82 (PCN: Primary Care Network)	5	5.1	4
1	SU	"Service implementation was limited by the mismatch between patient needs and what providers considered appropriate." p. xix	6	6.1	4
1	SU	"The same interviewee reported that before COVID-19 a higher proportion of service users were from ethnic minority communities, but post COVID-19 a lot more work was taking place in areas that are predominantly white British and wealthy." p. 25	5	5.2	4
1	SYS	"A variety of costs and savings (key area 10) issues were described by participants, including concerns about lack of support for 'additional costs' such as overheads, training and voluntary sector costs." p. 27	7	7.1	7
1	LW	"Training programmes provided to link workers differed. A high turnover of link workers was reported." p. xix	7	7.2	8
2	SYS	"The follow-on services that patients access are often underfunded or short term." p. v	7	7.2	7

Table S5: Characteristics of Included Studies

Author/Year	Study Type & Study Design	Aim	Setting/Sample Characteristics	Harms, Negative and Unintended Consequences
Al-Khudairy et al., 2022 [2]	Qualitative - Rapid systematic review and semi-structured interviews	To explore what evidence and evaluation methods are needed to assess the impact of link workers for individuals.	UK, England. Social prescribing programs. Link workers and stakeholders (N=25)	N=7 Health System (n=1), Equity and Equality-Related (n=2), Social and Institutional (n=1), Economic and Resource-Related (n=3)
Aughterson et al., 2020 [3]	Qualitative - Semi-structured interviews	To identify factors that help or hinder GPs in implementing social prescribing for mental health individuals.	UK. General practice. GPs (N=17)	N=5 Health (n=1), Health System (n=3), Social and Institutional (n=1)
Avon Wildlife Trust, 2021 [4]	Mixed-methods - Interviews and survey	To assess the impact of nature-based programs on psychosocial outcomes.	UK, England. Voluntary sector. Service users (N=1995)	N=9 Health (n=2), Health System (n=4), Equity and Equality-Related (n=1), Social and Institutional (n=2)
Baker, 2016 [5]	Mixed-methods - Semi-structured interviews, focus groups, survey, observations, document review	To examine the functioning and delivery of a pilot social prescribing program for people at risk of social isolation.	UK, North East England. Community organization and primary care collaboration. Stakeholders, service users, carers (N=48)	N=2 Health System (n=1), Economic and Resource-Related (n=1)
Beardmore, 2019 [6]	Qualitative - Semi-structured interviews	To explore link workers' experiences, career paths, and progression in the sector.	UK, South West England. Social prescribing services. Link workers (N=8)	N=8 Health (n=2), Health System (n=6)
Bertotti et al., 2020 [7]	Mixed-methods -	To assess the implementation,	UK, England (London). Primary	N=16 Health (n=2), Health System (n=7),

	Interviews, survey, process and economic evaluation	outcomes, and cost-effectiveness of social prescribing in Redbridge.	care and voluntary sector. Service users (N=182)	Human and Fundamental Rights (n=1), Acceptability and Adherence (n=2), Social and Institutional (n=2), Economic and Resource-Related (n=2)
Bertotti et al., 2017 [8]	Mixed-methods - Interviews, process and economic evaluation	To evaluate the impact, integration, and value for money of a social prescribing pilot.	UK, London (Waltham Forest). General practices and council. Service users (N=9)	N=23 Health (n=5), Health System (n=10), Acceptability and Adherence (n=2), Equity and Equality-Related (n=3), Social and Institutional (n=2), Economic and Resource-Related (n=1)
Bertotti et al., 2018 [9]	Mixed-methods - Realist evaluation (interviews, focus groups, survey)	To investigate which elements of a social prescribing pilot worked, for whom, and under what circumstances.	UK, London (City and Hackney). Primary care. Service users (N=7) and stakeholders from primary care and the voluntary sector	N=4 Health System (n=1), Equity and Equality-Related (n=1), Social and Institutional (n=1), Economic and Resource-Related (n=1)
Blickem et al., 2013 [10]	Qualitative - Focus groups, participatory workshops, interviews	To gather service user insights to inform development of a community referral intervention for long-term conditions.	UK, England (Greater Manchester). Community sector. Service users (N=40)	N=4 Health (n=2), Equity and Equality-Related (n=1), Social and Institutional (n=1)
Brandling & House, 2007 [11]	Qualitative - Semi-structured interviews and records evaluation	To explore the feasibility of conducting a multi-center randomized controlled trial for a social prescribing	UK, England (Keynsham and Bath/North East Somerset). Primary care. Service users and	N=4 Health System (n=2), Human and Fundamental Rights (n=2)

		service in primary care.	stakeholders (N=19)	
Brandling & House, 2009 [12]	Commentary	To discuss the meaning and context of social prescribing in general practice.	UK. Social prescribing in general practice.	N=2 Health System (n=1), Acceptability and Adherence (n=1)
Bu et al., 2024 (pre-print only) [13]	Quantitative - Records evaluation	To explore differences in demographics, referral reasons, contacts, and interventions in SP pathways.	UK. Social prescribing programs. Records of service users (N=160,000)	N=10 Health System (n=4), Acceptability and Adherence (n=1), Equity and Equality-Related (n=2), Economic and Resource-Related (n=3)
Bybee et al., 2024 [14]	Quantitative - Records evaluation	To examine differences in patient characteristics and referral processes among individuals visiting the emergency department with social needs and varying technology access.	USA, Utah. Emergency department. Service users N=453)	N=7 Health System (n=5), Acceptability and Adherence (n=1), Equity and Equality-Related (n=1)
Bywaters et al., 2011 [15]	Quantitative - Survey	To determine the extent of social care provision and develop a taxonomy of emergency department social care initiatives.	UK. Emergency departments. Social care teams (N=208)	N=6 Health System (n=4), Equity and Equality-Related (n=2)
Carnes et al., 2015 [16]	Report- Mixed-methods - Interviews, focus groups, field observation, evaluation of healthcare	To assess the effect of a social prescribing project on individuals, team awareness, and costs.	UK, England (London -City and Hackney). General practice and voluntary sector. Interviews with service users, stakeholders	N=4 Health (n=4)

	data and assessments		(N=19) and focus groups with stakeholders	
Carnes et al., 2017 [17]	Mixed-methods - Patient surveys with matched control groups, qualitative interviews	To investigate implementation and effects of a social prescribing service in general practice.	UK, London (City and Hackney). General practice. Service users (N=585)	N=1 Health System (n=1)
Christofides & Jewkes, 2010 [18]	Qualitative - In-depth interviews, focus group	To explore women's experiences of IPV screening in HIV testing and counseling services.	South Africa, (Johannesburg). HIV testing clinics. Service users (N=35)	N=7 Health (n=1), Health System (n=2), Acceptability and Adherence (n=1), Social and Institutional (n=3)
Dayson & Batty, 2020 [19]	Report-Qualitative - Case studies, interviews	To analyze the role and experiences of small providers in delivering social prescribing services.	UK, England (Rotherham). Community organizations. Case studies and interviews with providers and service users (N=30+)	N=5 Health (n=1), Social and Institutional (n=3), Economic and Resource-Related (n=1)
Dayson & Damm, 2020 [20]	Report - Quantitative - Service data review	To review well-being outcomes for people with long-term conditions involved in a social prescribing service.	UK, England (Rotherham). Voluntary and community sector. Service users (N=878)	N=2 Economic and Resource-Related (n=2)
Dayson et al., 2013 [21]	Report - Mixed-methods - Summary report	To evaluate a social prescribing pilot project and well-being outcomes for service users.	UK, England (Rotherham). General practice. Service users (N=878)	N=7 Health (n=2), Equity and Equality-Related (n=3), Social and Institutional (n=1), Economic and Resource-Related (n=1)

Dayson et al., 2019 [22]	Qualitative - Comparative analysis, interviews	To compare two social prescribing interventions with different financing approaches.	UK, Northern England. Social prescribing programs. Stakeholders (N=98)	N=2 Social and Institutional (n=1), Economic and Resource-Related (n=1)
Dickens et al., 2011 [23]	Quantitative - Controlled trial	To examine the effectiveness of a community-based mentoring service for socially isolated older people.	UK. Voluntary sector. Service users (N=374)	N=1 Social and Institutional (n=1)
Din et al., 2015 [24]	Qualitative - Semi-structured group interviews	To explore health professionals' perceptions of exercise referral and physical activity promotion.	UK, Wales. General practice. Health professionals (N=46)	N=10 Health System (n=2), Acceptability and Adherence (n=4), Equity and Equality-Related (n=3), Economic and Resource-Related (n=1)
Ell et al., 2018 [25]	Quantitative - Randomized controlled trial	To evaluate the effectiveness of a bilingual community health worker intervention for depression and self-care of individuals.	USA, Los Angeles. Primary care. Service users (N=348)	N=3 Health (n=1), Acceptability and Adherence (n=1), Economic and Resource-Related (n=1)
Elston et al., 2019 [26]	Quantitative - Questionnaires, records evaluation	To evaluate if a holistic link-worker intervention improves well-being and frailty in older people and if it impacts healthcare and socialcare usage.	UK, South West England. Primary care. Service users (N=86)	N=5 Health (n=1), Health System (n=1), Economic and Resource-Related (n=3)
Farenden, 2015 [27]	Report - Mixed-methods - interviews, surveys	To evaluate a community navigation social prescribing pilot.	UK, England (Brighton & Hove). Primary care. Service users (N=393) and surveys for	N=19 Health (n=4), Health System (n=5), Acceptability and Adherence (n=5), Equity and Equality-Related (n=2),

			volunteers and GPs	Economic and Resource-Related (n=3)
Faulkner, 2004 [28]	Qualitative - Semi-structured interviews	To describe and analyze the perceived effectiveness and barriers of a volunteer patient support service for psychosocial needs.	UK, England (Doncaster). General practice. Service users (N=20)	N=4 Health System (n=1), Social and Institutional (n=3)
Fixsen & Barrett, 2022 [29]	Qualitative - Interviews	To examine challenges and opportunities for delivering green social prescribing during and after COVID-19.	UK, Scotland and North East England. Nature-based social prescribing programs. Stakeholders (N=28)	N=7 Health System (n=1), Acceptability and Adherence (n=1), Equity and Equality-Related (n=1), Social and Institutional (n=2), Economic and Resource-Related (n=2)
Fixsen et al., 2021 [30]	Qualitative - Interviews	To compare responses of three social prescribing schemes in Scotland to the COVID-19 pandemic.	UK, Scotland. Social prescribing programs. Stakeholders (N=23)	N=8 Health System (n=1), Human and Fundamental Rights (n=1), Acceptability and Adherence (n=1), Equity and Equality-Related (n=2), Social and Institutional (n=3)
Foster et al., 2021 [31]	Mixed-methods - Pre-post analysis, social return on investment analysis, semi-structured interviews	To assess the impact of a national social prescribing program on loneliness.	UK. Community organization. Service users, link workers and volunteers (N=60)	N=5 Health (n=1), Health System (n=1), Acceptability and Adherence (n=2), Economic and Resource-Related (n=1)

Frerichs et al., 2020 [32]	Qualitative - Semi-structured interviews	To explore factors enabling or hindering participation in a community navigation intervention for people experiencing severe depression and/or anxiety.	UK. Primary care. Service users (N=19)	N=14 Health (n=6), Human and Fundamental Rights (n=1), Acceptability and Adherence (n=1), Equity and Equality-Related (n=1), Social and Institutional (n=4), Economic and Resource-Related (n=1)
Frostick & Bertotti, 2021 [33]	Qualitative - In-dept interviews, focus groups	To identify training, skills, and experience needed for link workers in primary care.	UK, England (London and South East England). Primary care. Link workers from three different SP schemes (N=13)	N=4 Health (n=1), Health System (n=3)
Fullwood, 2018 [34]	Report- Mixed-methods- record evaluation, interviews	To evaluate a personalized integrated care program for older adults.	UK. Primary care. Service users (N=2071)	N=7 Health (n=1), Acceptability and Adherence (n=5), Economic and Resource-Related (n=1)
Galvin et al., 2000 [35]	Mixed Methods - interviews, focus groups, survey	To assess the impact of Citizens Advice Bureaux in primary care from multiple perspectives.	UK, Southern England. Primary care. Service users, stakeholders (N=12)	N=2 Health System (n=1), Acceptability and Adherence (n=1)
Galway et al., 2019 [36]	Qualitative - stakeholder workshops, interviews	To explore the acceptability and value of digital social prescribing for suicide bereavement support among various stakeholders.	UK, Northern Ireland. Community and voluntary sector. Stakeholders (N=30)	N=3 Health (n=1), Human and Fundamental Rights (n=1), Equity and Equality-Related (n=1)
Garside et al., 2020 [37]	Report - Mixed-methods -	To evaluate the effectiveness and provision of nature-	UK, Northern England. Nature-based social	N=48 Health (n=11), Health System (n=14), Human and

	Review, case studies, interviews	based social prescribing programs for people with diagnosed mental health conditions.	prescribing programs. Interviews with stakeholders (N=32)	Fundamental Rights (n=1), Acceptability and Adherence (n=8), Equity and Equality-Related (n=1), Social and Institutional (n=3), Economic and Resource-Related (n=9), Ecological (n=1)
Gibson et al., 2021 [38]	Qualitative - Ethnographic research - interviews, observation, photo-elicitation interviews	To explore social contexts in which social prescribing for people with Type 2 Diabetes is delivered and its implications on health inequalities.	UK, Northern England. Primary care. Service users and family members (N=19)	N=8 Health (n=1), Acceptability and Adherence (n=1), Equity and Equality-Related (n=2), Social and Institutional (n=3), Economic and Resource-Related (n=1)
Giebel et al., 2021 [39]	Quantitative - pre and post-assessment of well-being scores	To evaluate a socially prescribed community service for people with dementia and their carers.	UK, North West England. Community sector. People with dementia and carers (N=25)	N=1 Economic and Resource-Related (n=1)
Grant et al., 2000 [40]	Quantitative - Randomized controlled trial	To compare psychosocial outcomes and resource use between individuals referred to a referral service and those receiving usual GP care.	UK, England. Primary care. Service users (N=161)	N=2 Economic and Resource-Related (n=2)
Griffith et al., 2023 [41]	Qualitative - Ethnographic research - semi-structured interviews, observation, focus-groups	To identify factors shaping link worker practices and context of service delivery in social prescribing schemes for people with long-term conditions.	UK, Northern England. Community organization. Link workers (N=20)	N=7 Health (n=2), Health System (n=3), Social and Institutional (n=2)

Griffiths et al., 2023 [42]	Qualitative - Semi-structured interviews	To explore link workers' perspectives on social prescribing service delivery in the NHS.	UK, Central England. Primary care. Link workers (N=18)	N=11 Health (n=3), Health System (n=5), Acceptability and Adherence (n=1), Equity and Equality-Related (n=1), Social and Institutional (n=1)
Hamlin et al., 2016 [43]	Quantitative - telephone survey	To assess the long-term effectiveness of a primary care physical activity intervention.	New Zealand. Primary care. Green Prescription participants (N=147)	N=5 Health (n=1), Health System (n=1), Equity and Equality-Related (n=2), Social and Institutional (n=1)
Hanlon et al., 2021 [44]	Qualitative - semi-structured interviews	To explore how Self-Determination Theory explains impacts of a social prescribing intervention.	UK, Scotland (Glasgow). General practice. Individuals (N=12)	N=9 Health (n=4), Health System (n=1), Equity and Equality-Related (n=3), Social and Institutional (n=1)
Hazeldine et al., 2021 [45]	Qualitative - interviews	To explore link workers' experiences and acceptability of an SP implementation.	UK, South West England. Primary care. Link workers, managers, counsellor (N=14)	N=7 Health (n=3), Health System (n=1), Equity and Equality-Related (n=1), Social and Institutional (n=2)
Heijnders & Meijs, 2018 [46]	Qualitative - Semi-structured interviews	To explore SP pathways and factors influencing the perception of social participation among individuals with psychosocial problems.	Netherlands, Nieuwegein. Primary care. Individuals (N=10)	N=3 Health System (n=1), Acceptability and Adherence (n=2)
Holding et al., 2020 [47]	Qualitative - Interviews	To Investigate challenges and needed resources in the delivery of a national social prescribing service to reduce loneliness.	UK. Community organization. Link workers, volunteers (N=28)	N=10 Health (n=2), Health System (n=4), Acceptability and Adherence (n=1), Economic and Resource-Related (n=3)

Innovation Unit, 2016 [48]	Report - Mixed-methods - Interviews, workshops, record evaluation	To evaluate the Wigan Community Link Worker Service.	UK, England (Wigan). Primary care. Stakeholders and service users (N=41)	N=15 Health System (n=7), Acceptability and Adherence (n=1), Equity and Equality-Related (n=3), Social and Institutional (n=1), Economic and Resource-Related (n=3)
Isaacs et al., 2007 [49]	Quantitative - Randomized controlled trial	To compare effectiveness and cost-effectiveness of different exercise referral programs for people with at least one cardiovascular risk factor.	UK, Engand (London -Barnet). Community organization. Service users of leisure centers (n=317), walking groups (n=311) and a control-group receiving exercise-advice only (n=315).	N=10 Health (n=5), Health System (n=1), Acceptability and Adherence (n=1), Equity and Equality-Related (n=2), Social and Institutional (n=1)
Islam, 2019 [50]	Report - Mixed-methods - record data	To evaluate the SP service of Bromley by Bow Centre	UK, England (London). Community organization. Service users from 6 GPs (583 referrals)	N=3 Health (n=1), Human and Fundamental Rights (n=1), Acceptability and Adherence (n=1)
Healthwatch Islington, 2019 [51]	Report- Mixed-methods - Survey, interviews, focus groups	To evaluate social prescribing and navigation services in Islington.	UK, England (London - Islington). Primary care. Service users (N=154)	N=12 Health System (n=2), Human and Fundamental Rights (n=2), Acceptability and Adherence (n=2), Equity and Equality-Related (n=1), Social and Institutional (n=5)
Jones & Lynch, 2019 [52]	Mixed-methods - Focus groups,	To evaluate the SPICE Time Credit Social Prescribing Pilot.	UK, Wales. Community organization. Practice staff,	N=10 Health (n=2), Health System (n=3), Acceptability and Adherence (n=2), Economic and

	interviews, surveys		service users (N=12)	Resource-Related (n=3)
Kellezi et al., 2019 [53]	Mixed-methods - Survey, interviews	To assess the impact of SP schemes on healthcare usage for people with chronic conditions experiencing loneliness.	UK, England (East Midlands). General practice. Semi-structured interviews with GPs, healthcare providers and service users (N=35) and survey among 630 individuals.	N=4 Health (n=2), Health System (n=1), Social and Institutional (n=1)
Kellezi et al., 2020 [54]	Qualitative - Co-production design (stakeholder discussions)	To identify barriers and facilitators for using social prescribing with migrants.	UK. Social prescribing programs for migrants. Stakeholders, migrants, link workers, academics (N=40)	N=12 Health (n=2), Health System (n=2), Acceptability and Adherence (n=1), Equity and Equality-Related (n=2), Social and Institutional (n=5)
Kensington & Chelsea Social Council & NHS West London CCG, 2018 [55]	Report - Mixed-methods - Survey, interviews, social Return on Investment analysis	To calculate the social return on investment of a self-care social prescribing model.	UK, England (London). General practices. Service users, GPs and other stakeholders (N=255)	N=9 Health System (n=2), Acceptability and Adherence (n=1), Equity and Equality-Related (n=1), Social and Institutional (n=4), Economic and Resource-Related (n=1)
Khan et al., 2023 [56]	Quantitative - Evaluation of patient records	To investigate the feasibility of identifying health inequities and referral decline in SP through primary care records.	UK, Northwest England. General practices. Individuals (N=3086)	N=3 Equity and Equality-Related (n=3)
Kharicha et al., 2017 [57]	Qualitative - Interviews	To explore service users views on primary care and	UK, England (London and semi-rural	N=7 Human and Fundamental Rights (n=2), Acceptability

		community interventions for loneliness.	county). Primary care. Service users (N=28)	and Adherence (n=3), Social and Institutional (n=2)
Kiely et al., 2021 [58]	Mixed-methods - Structured interviews and survey	To examine the effectiveness of primary care-based link workers for people with multimorbidity.	UK, Glasgow. General practice. Individuals, link worker, GPs (N=14)	N=1 Economic and Resource-Related (n=1)
Kimberlee, 2016 [59]	Report - Mixed-methods - record evaluation	To evaluate the effectiveness of a SP pilot across Gloucestershire.	UK, England (Gloucestershire). General practices. Individuals (N=2047)	N=8 Health (n=1), Health System (n=3), Human and Fundamental Rights (n=1), Equity and Equality-Related (n=1), Economic and Resource-Related (n=2)
Longwill, 2014 [60]	Report - Mixed-methods - Interviews, observations, survey, record evaluation	To evaluate the impact of social risk screening and referral interventions in safety-net settings.	UK, England (London - Hackney). Primary care. Service users (N=1089)	N=9 Health System (n=2), Acceptability and Adherence (n=2), Equity and Equality-Related (n=3), Social and Institutional (n=2)
Loo, 2023 [61]	Thesis - Mixed-methods - Interviews, record evaluation	To explore the impact of social risk screening and referral interventions in safety-net settings.	USA, Boston. Primary care and breast cancer clinics. Individuals, navigators, staff (N=70144, 17 interviews)	N=14 Health (n=3), Health System (n=1), Human and Fundamental Rights (n=2), Acceptability and Adherence (n=4), Equity and Equality-Related (n=2), Economic and Resource-Related (n=2)
Lowe et al., 2019 [62]	Qualitative - Case study, interviews	To examine how Social Impact Bond financing influenced	UK, Northern England. Voluntary sector. Stakeholders,	N=5 Acceptability and Adherence (n=2), Equity and Equality-Related (n=2),

		actors in a social prescribing program.	service users (N=22)	Economic and Resource-Related (n=1)
McHale et al., 2020 [63]	Qualitative - Focus groups, interviews	To explore professional perspectives on Green Health Partnerships and referral pathways.	UK, Scotland. Social prescribing programs. Health board members (N=55)	N=3 Equity and Equality-Related (n=2), Economic and Resource-Related (n=1)
McLeish & Redshaw, 2015 [64]	Qualitative - Semi-structured interviews	To explore experiences of using peer support during pregnancy and early parenthood.	UK, England. Voluntary sector. Volunteers, service users (N=89)	N=8 Health (n=3), Acceptability and Adherence (n=1), Social and Institutional (n=3), Economic and Resource-Related (n=1)
McLeish & Redshaw, 2016 [65]	Qualitative - Interviews	To explore experiences of women living with HIV receiving or giving peer support during pregnancy.	UK, England (London). Voluntary sector. Service users (N=12)	N=3 Health (n=2), Human and Fundamental Rights (n=1)
McLeish, 2017 [66]	Qualitative - Semi-structured interviews	To explore the experience of volunteers providing support for disadvantaged mothers.	UK, England. Voluntary sector. Volunteers (N=38)	N=11 Health (n=7), Acceptability and Adherence (n=2), Social and Institutional (n=1), Economic and Resource-Related (n=1)
Mistry et al., 2023 [67]	Mixed-methods - Interviews, record evaluation	To explore the feasibility and acceptance of bilingual community navigators in primary care.	Australia, Sydney. General practice. Individuals, navigators, stakeholders (N=95, 16 interviews)	N=3 Health System (n=1), Equity and Equality-Related (n=1), Economic and Resource-Related (n=1)
Moffatt et al., 2023 [68]	Report - Mixed-methods - Quasi-experimental	To evaluate the impact and costs of a link worker social prescribing intervention for	UK, North East England. Primary care. Individuals, link workers, stakeholders	N=20 Health (n=3), Health System (n=4), Human and Fundamental Rights (n=2), Acceptability

	evaluation, cost-effectiveness evaluation, interviews, Record evaluation	adults with type 2 diabetes.	(N=8400, 44 interviews)	and Adherence (n=6), Equity and Equality-Related (n=2), Economic and Resource-Related (n=3)
Moore et al., 2023 [69]	Qualitative - Semi-structured interviews	To explore self-perceived professional identity among social prescribing link workers.	UK, England. Social prescribing programs. Link workers (N=13)	N=12 Health (n=1), Health System (n=4), Acceptability and Adherence (n=1), Equity and Equality-Related (n=1), Social and Institutional (n=4), Economic and Resource-Related (n=1)
Morris et al., 2022 [70]	Qualitative - Interviews	To analyze adaptations of a social prescribing service during the COVID-19 pandemic.	UK, North East England. Voluntary and community sector. Service users, link workers, stakeholders (N=57)	N=7 Health (n=1), Acceptability and Adherence (n=3), Equity and Equality-Related (n=3)
National Association of Link Workers, 2019 [71]	Quantitative - Survey	To gain insight into link workers' knowledge, skills, experiences, and support needs.	UK. Social prescribing programs. Link workers (N=105)	N=5 Health (n=2), Health System (n=1), Acceptability and Adherence (n=1), Social and Institutional (n=1)
NHS England, 2020 [72]	Report	To provide a guide for SP implementation.	UK. Social prescribing programs.	N=1 Health System (n=1)
O'Brien et al., 2010 [73]	Mixed-methods - Interviews and questionnaires	To identify motivations, barriers, and benefits of voluntary work in the environment.	UK, Northern England and Southern Scotland. Voluntary sector. Volunteers (N=88)	N=5 Health (n=3), Equity and Equality-Related (n=1), Social and Institutional (n=1)

Palmer & Sango, 2017 [74]	Report - Mixed-methods - Interviews, questionnaire	To evaluate the benefits and limitations of a social prescribing pilot in Bexley.	UK, England (London -Bexley). General practices. Participants (N=245)	N=2 Equity and Equality-Related (n=2)
Patel et al., 2021 [75]	Qualitative - Survey	To identify opportunities and challenges for digital social prescribing in mental health.	UK. Digital social prescribing programs. Nonexpert and expert participants (N=44)	N=18 Health (n=1) Health System (n=1), Human and Fundamental Rights (n=3), Acceptability and Adherence (n=5), Equity and Equality-Related (n=3), Social and Institutional (n=4), Economic and Resource-Related (n=1)
Pescheny et al., 2018 [76]	Qualitative - Semi-structured interviews	To explore experiences and views on uptake and adherence to social prescribing.	UK, East of England (Luton). General practice. Service users, navigators, GPs (N=15)	N=5 Human and Fundamental Rights (n=1), Acceptability and Adherence (n=4)
Pescheny et al., 2021 [77]	Quantitative - Questionnaires	To analyze changes in mental well-being for people experiencing loneliness and having certain risk factors after participating in SP.	UK, Luton. Primary care. Service users (N=63)	N=2 Health System (n=1), Acceptability and Adherence (n=1)
Pollard et al., 2023 [78]	Qualitative - Ethnographic exploration	To explore delivery and gain insight into experiences of link workers as part of a social prescribing intervention.	UK, Northern England. General practice. Link workers, service users (N=39)	N=10 Health (n=1), Health System (n=3), Human and Fundamental Rights (n=1), Equity and Equality-Related (n=2), Social and Institutional (n=2), Economic and

				Resource-Related (n=1)
Pons-Vigues et al., 2019 [79]	Qualitative - Interviews, group discussions	To evaluate the implementation and development of a health promotion intervention.	Spain, multiple regions. Primary care. Service users, Stakeholders (N=94)	N=5 Health System (n=2), Acceptability and Adherence (n=1), Equity and Equality-Related (n=1), Economic and Resource-Related (n=1)
Poole & Huxely, 2024 [80]	Commentary	To critically discuss the evidence base for social prescribing in mental health.	UK. Social prescribing programs in mental health.	N=10 Health (n=1), Health System (n=4), Acceptability and Adherence (n=1), Equity and Equality-Related (n=2), Social and Institutional (n=1), Economic and Resource-Related (n=1)
Rathbone et al., 2023 [81]	Qualitative - Semi-structured interviews	To explore community pharmacists' views on implementing social prescribing in pharmacies.	UK, Northern England. Pharmacies. Stakeholders (N=11)	N=5 Health System (n=2), Human and Fundamental Rights (n=1), Economic and Resource-Related (n=2)
Rhodes & Bell, 2021 [82]	Qualitative - Semi-structured interviews	To explore challenges and experiences of social prescribing link workers.	UK, England (London). Primary care and voluntary sector. Link workers (N=9)	N=8 Health (n=3), Health System (n=3), Social and Institutional (n=2)
Robinson et al., 2020 [83]	Qualitative - Survey	To explore awareness, constraints and opportunities of delivering nature-based green prescribing in the UK.	UK. General practice, community organizations. GPs, NBOs (N=303)	N=3 Health System (n=1), Social and Institutional (n=1), Economic and Resource-Related (n=1)

Rowe et al., 2020 [84]	Report - Mixed-methods - Survey, records evaluation	To evaluate a SP program in the UK in the time period of 2019-2020.	UK. Social prescribing programs. Service users (N=918)	N=13 Health (n=2), Health System (n=3), Acceptability and Adherence (n=2), Equity and Equality-Related (n=4), Social and Institutional (n=2)
Scarpetti et al., 2024 [85]	Quantitative - Survey	To compare social prescribing approaches across twelve high-income countries.	Australia, Austria, Canada, England, Finland, Germany, Portugal, Slovakia, Slovenia, Netherlands, USA, Wales. Social prescribing programs. Country experts (N=12)	N=5 Health System (n=1), Acceptability and Adherence (n=2), Equity and Equality-Related (n=1), Economic and Resource-Related (n=1)
Schmidt et al., 2008 [86]	Mixed-methods - Questionnaire, interviews	To analyze sociodemographic factors of women engaging in exercise referral schemes in deprived areas.	Netherlands, The Hague. Community organization. Service users (N=523) and 38 interviews	N=9 Health (n=2), Acceptability and Adherence (n=2), Equity and Equality-Related (n=1), Social and Institutional (n=2), Economic and Resource-Related (n=2)
Simpson et al., 2020 [87]	Qualitative - Interviews	To investigate barriers and resources for people with motor-neurone disease participating in SP.	UK. Community organization. Service users, link workers (N=18)	N=8 Health (n=2), Health System (n=1), Human and Fundamental Rights (n=1), Equity and Equality-Related (n=1), Social and Institutional (n=2), Economic and Resource-Related (n=1)

Skivington et al., 2018 [88]	Qualitative - Semi-structured interviews	To investigate factors relevant for implementing SP programs.	UK, Scotland (Glasgow). Community organizations. Link workers, stakeholders (N=36)	N=4 Health System (n=1), Acceptability and Adherence (n=1), Economic and Resource-Related (n=2)
South et al., 2008 [89]	Qualitative - Interviews	To explore the concept and value of social prescribing as a public health initiative.	UK. General practice. Service users, health professionals (N=18)	N=5 Health System (n=3), Acceptability and Adherence (n=1), Equity and Equality-Related (n=1)
Southby & Gamsu, 2018 [90]	Qualitative - Case study, interviews, focus group	To investigate relevant factors that facilitate or hinder the collaboration of GP and the voluntary/community sector.	UK, Northern England. General practice, voluntary and community organizations. Stakeholders (N=18)	N=6 Human and Fundamental Rights (n=1), Acceptability and Adherence (n=3), Social and Institutional (n=1), Economic and Resource-Related (n=1)
Strachan et al., 2007 [91]	Quantitative - Survey	To assess health and well-being improvements of participants from a community health intervention.	UK, Scotland. Community organization. Service users (N=90)	N=3 Health System (n=1), Acceptability and Adherence (n=1), Equity and Equality-Related (n=1)
Stuart et al., 2022 [92]	Qualitative - Semi-structured interviews	To identify barriers preventing lonely or isolated people from joining groups for health and well-being.	UK. Community organization. Service users (N=11)	N=12 Health (n=4), Health System (n=1), Human and Fundamental Rights (n=1), Acceptability and Adherence (n=4), Social and Institutional (n=2)
Sumner et al., 2020 [93]	Quantitative - Records evaluation	To analyze factors associated with attendance, engagement, and well-being change in arts on prescription.	UK, South West England. Community organization. Service users (N=1297)	N=4 Health (n=1), Equity and Equality-Related (n=3)

Todd, 2017 [94]	Thesis - Qualitative - Interviews	To explore how museum programs for older people enhance well-being and reduce social isolation.	UK, England (London and Kent). Community Organization. Service users (N=12)	N=10 Health (n=1), Human and Fundamental Rights (n=2), Equity and Equality-Related (n=3), Social and Institutional (n=4)
Tran, 2009 [95]	Report- Mixed-methods - case studies, interviews	To evaluate Mental Health Advocacy and Support Project for Service users of Chinese ethnicity.	UK, England (London). Primary care. Service users (N=40)	N=2 Acceptability and Adherence (n=1), Equity and Equality-Related (n=1)
Vogelpoel & Jarrold, 2014 [96]	Mixed-methods - Interviews, questionnaires	To assess the benefits of a social prescribing service for older people with sensory impairments.	UK, England (Rotherham). Community organization. Service users (N=12)	N=2 Health (n=1), Social and Institutional (n=1)
Westlake et al., 2024 [97]	Qualitative - interviews, records evaluation	To explore the concept of “holding” within SP.	UK, England. General practices. Service users and stakeholders (N=154)	N=22 Health (n=8), Health System (n=10), Acceptability and Adherence (n=3), Economic and Resource-Related (n=1)
White & Kinsella, 2010 [98]	Report - Mixed-methods - Interviews, record evaluation	To evaluate the Bradford Health Trainer and Social Prescribing Service.	UK, England (Bradford). General practices. Service users, health trainers (N=22)	N=6 Health (n=1), Health System (n=4), Acceptability and Adherence (n=1)
Whitelaw et al., 2017 [99]	Qualitative - Interviews, case study	To evaluate the implementation of a SP initiative in primary care.	UK, Scotland. Primary care. Stakeholders (N=30)	N=5 Health (n=1), Health System (n=1), Human and Fundamental Rights (n=1), Acceptability and Adherence (n=1), Economic and Resource-Related (n=1)

Wildman et al., 2019a [100]	Qualitative - Interviews, focus groups	To explore link workers' definitions of their role and skills needed for social prescribing.	UK, North East England. Voluntary sector. Link workers (N=26)	N=11 Health (n=1), Health System (n=5), Acceptability and Adherence (n=1), Equity and Equality-Related (n=2), Social and Institutional (n=2)
Wildman et al., 2019b [101]	Qualitative - Semi-structured interviews	To explore the experiences of social prescribing among people with long-term conditions.	UK, North East England. Voluntary sector. Service users (N=24)	N=6 Health System (n=4), Acceptability and Adherence (n=1), Social and Institutional (n=1)
Wilson et al., 2009 [102]	Report - Mixed-methods - Interviews, focus group, observations, questionnaire	To evaluate a greenspace and conservation on referral service for clients that uses mental health services.	UK, Scotland. Community organization. Service users (N=125)	N=10 Health (n=5), Health System (n=1), Acceptability and Adherence (n=3), Social and Institutional (n=1)
Woodall et al., 2018 [103]	Mixed-methods - Interviews, Focus group, Survey	To evaluate the effectiveness and process of a social prescribing service.	UK, Northern England. Social prescribing programs. Service users (N=342), interviews (N=26)	N=6 Health System (n=2), Equity and Equality-Related (n=4)
Wormald & Ingle, 2004 [104]	Qualitative - Focus groups	To explore individuals' perceptions of GP exercise referral schemes.	UK, England (North Yorkshire). Community organizations. Service users (N=30)	N=4 Acceptability and Adherence (n=3), Equity and Equality-Related (n=1)
Wormald et al., 2006 [105]	Qualitative - Focus groups	To explore participants' perceptions of a lifestyle approach to promote physical activity.	UK, England (Kingston-upon-Hull) Community organization. Service users (N=16)	N=5 Health System (n=1), Acceptability and Adherence (n=3), Social and Institutional (n=1)
Yichao et al., 2022 [106]	Qualitative - Semi-structured Interviews,	To explore challenges in implementing community-led	UK, England (Bradford, Walsall). Community and	N=13 Health System (n=3), Acceptability and Adherence (n=3), Social and Institutional

	literature review	nature-based solutions for health and well-being.	voluntary organizations. Stakeholders (N=26)	(n=2), Economic and Resource-Related (n=4), Ecological (n=1)

Table S6: List of Studies Excluded at the Full-Text Screening Stage from the Database Search

Author	Title	Reason for Exclusion
Barlett-Prescott et al., 2004	Health promotion referrals in an urban clinic: Removing financial barriers influences physician but not patient behavior	No SP Intervention
Charlson et al., 2008	Outcomes of community-based social service interventions in homebound elders	No SP Intervention
Choi et al., 2016	Sources of referral in student financial counseling	No SP Intervention
Cooper et al., 2022	Effectiveness and active ingredients of social prescribing interventions targeting mental health: A systematic review	No information on HNUCs
Cooper et al., 2023	Service user perspectives on social prescribing services for mental health in the UK: A systematic review	No information on HNUCs
Costa et al., 2024	Social prescribing for older adults in mainland Portugal: perceptions and future prospects	No information on HNUCs
Dabbs, 2024	Social prescribing: Community power and the community paradigm	No information on HNUCs
DeLuca et al., 2021	Reducing covid-19 health inequities through identification of health-related social needs	No information on HNUCs
Elliott et al., 2022	Exploring how and why social prescribing evaluations work: A realist review	No information on HNUCs
Evans, 2023	Assessing online faculty likelihood to refer students to university mental health services	No information on HNUCs

Evers et al., 2024	Theories used to develop or evaluate social prescribing in studies: A scoping review	No information on HNUCs
Featherstone et al., 2022	Health and wellbeing outcomes and social prescribing pathways in community-based support for autistic adults: A systematic mapping review of reviews	No information on HNUCs
Floyd, 2019	Implementing a social determinants of health screening tool at a community health clinic	No information on HNUCs
Ghezelayagh et al., 2023	Incorporation of a social needs screening tool with subsequent social work support in a large-volume gynecologic oncology clinic	No SP Intervention
Gomez et al., 2012	Project prescription for hope (RXH): Trauma surgeons and community aligned to reduce injury recidivism caused by violence	No information on HNUCs
Hodgson et al., 2021	Integrating primary care and social services for older adults with multimorbidity: Policy implications	No information on HNUCs
Howarth et al., 2020	What is the evidence for the impact of gardens and gardening on health and well-being: A scoping review and evidence-based logic model to guide healthcare strategy decision making on the use of gardening approaches as a social prescription	No information on HNUCs
Jones et al., 2023	Social issues, crisis, and care coordination: First responders experience responding to people affected by methamphetamines	No SP Intervention

Kaur et al., 2009	Interventions that can reduce inappropriate prescribing in the elderly: A systematic review	No SP Intervention
Kiely et al., 2022	Effect of social prescribing link workers on health outcomes and costs for adults in primary care and community settings: A systematic review	No information on HNUCs
Lee et al., 2023	Global trends in social prescribing: Web-based crawling approach	No information on HNUCs
Levy et al., 2021	From pandemic response to portable population health: A formative evaluation of the Detroit Mobile Health Unit program	No information on HNUCs
Lindsey et al., 2021	Social prescribing in community pharmacy: A systematic review and thematic synthesis	No information on HNUCs
Mann et al., 2017	A life less lonely: The state of the art in interventions to reduce loneliness in people with mental health problems	No information on HNUCs
Menhas et al., 2024	Does nature-based social prescription improve mental health outcomes? A systematic review and meta-analysis	No information on HNUCs
Moffatt et al., 2017	Link worker social prescribing to improve health and well-being for people with long-term conditions: Qualitative study of service user perceptions	No information on HNUCs
Mottershead, 2022	The social prescribing of psychosocial interventions in the treatment of addictions and substance use disorders with military veterans: A reclamation of identity and belonging	No information on HNUCs

Napierala et al., 2022	Social prescribing: Systematic review of the effectiveness of psychosocial community referral interventions in primary care.	No information on HNUCs
Nguyen et al., 2022	Nature prescriptions: A scoping review with a nested meta-analysis	No information on HNUCs
Nguyen et al., 2022	Effect of nature prescriptions on cardiometabolic and mental health, and physical activity: A systematic review	No information on HNUCs
Orellana et al., 2020	Day centres for older people - attender characteristics, access routes and outcomes of regular attendance: Findings of exploratory mixed methods case study research	No SP Intervention
Oster et al., 2023	Models of social prescribing to address non-medical needs in adults: A scoping review	No information on HNUCs
O'Sullivan et al., 2024	The effectiveness of social prescribing in the management of long-term conditions in community-based adults: A systematic review and meta-analysis	No information on HNUCs
Pescheny et al., 2018	Facilitators and barriers of implementing and delivering social prescribing services: A systematic review	No information on HNUCs
Pilkington et al., 2017	Searching for real-world effectiveness of health care innovations: Scoping study of social prescribing for diabetes	No information on HNUCs
Piraux et al., 2024	The community pharmacist as an independent prescriber: A scoping review	No SP Intervention
Reinhardt et al., 2021	Understanding loneliness: A systematic review of the	No information on HNUCs

	impact of social prescribing initiatives on loneliness	
Roberts et al., 2022	The role of social prescribers in wales: a consensus methods study	No information on HNUCs
Roth et al., 2023	Evaluation of an integrated intervention to address clinical care and social needs among patients with type 2 diabetes	No SP Intervention
Russell et al., 2010	A randomized controlled trial of a multifactorial falls prevention intervention for older fallers presenting to emergency departments	No SP Intervention
Sandhu et al., 2022	Intervention components of link worker social prescribing programmes: A scoping review	No information on HNUCs
Savage et al., 2020	Reply to social prescribing: creating pathways towards better health and wellness	No information on HNUCs
Sonke et al., 2023	Social prescribing outcomes: A mapping review of the evidence from 13 countries to identify key common outcomes	No information on HNUCs
Steeg et al., 2023	Social services utilisation and referrals after seeking help from health services for self-harm: A systematic review and narrative synthesis.	No SP Intervention
Thomas et al., 2022	Social prescribing of nature therapy for adults with mental illness living in the community: A scoping review of peer-reviewed international evidence.	No information on HNUCs
Thomson et al., 2020	Art, nature and mental health: assessing the biopsychosocial effects of a 'Creative Green Prescription' museum programme involving	No information on HNUCs

	horticulture, artmaking and collections	
Tully et al., 2022	Screening and referral for social determinants of health: maternity patient and health care team perspectives	No SP Intervention
Wahlbeck et al., 2017	Interventions to mitigate the effects of poverty and inequality on mental health	No information on HNUCs
Wallace et al., 2021	Using consensus methods to develop a social prescribing learning needs framework for practitioners in wales	No information on HNUCs
Wang et al., 2022	Horticultural therapy for general health in the older adults: A systematic review and meta-analysis	No information on HNUCs
Wildman, 2023	Impact of a link worker social prescribing intervention on non-elective admitted patient care costs: A quasi-experimental study	No information on HNUCs
Wittich et al., 2014	An adapted adult day centre for older adults with sensory impairment	No SP Intervention
Wu et al., 2023	Telephone-based social health screening by pharmacists in the nonadherent medicare population	No SP Intervention

Table S7: Preferred Reporting Items for Systematic reviews and Meta-Analyses extension for Scoping Reviews (PRISMA-ScR) Checklist [107]

SECTION	ITEM	PRISMA-ScR CHECKLIST ITEM	REPORTED ON PAGE #
**TITLE**			
Title	1	Identify the report as a scoping review.	1-2
**ABSTRACT**			
Structured summary	2	Provide a structured summary that includes (as applicable): background, objectives, eligibility criteria, sources of evidence, charting methods, results, and conclusions that relate to the review questions and objectives.	1-2
**INTRODUCTION**			
Rationale	3	Describe the rationale for the review in the context of what is already known. Explain why the review questions/objectives lend themselves to a scoping review approach.	2-3
Objectives	4	Provide an explicit statement of the questions and objectives being addressed with reference to their key elements (e.g., population or participants, concepts, and context) or other relevant key elements used to conceptualize the review questions and/or objectives.	3
**METHODS**			
Protocol and registration	5	Indicate whether a review protocol exists; state if and where it can be accessed (e.g., a Web address); and if available, provide registration information, including the registration number.	6
Eligibility criteria	6	Specify characteristics of the sources of evidence used as eligibility criteria (e.g., years considered, language, and publication status), and provide a rationale.	5
Information sources*	7	Describe all information sources in the search (e.g., databases with dates of coverage and contact with authors to identify additional sources), as well as the date the most recent search was executed.	5
Search	8	Present the full electronic search strategy for at least 1 database, including any limits used, such that it could be repeated.	Table S2.1-S2.3

SECTION	ITEM	PRISMA-ScR CHECKLIST ITEM	REPORTED ON PAGE #
Selection of sources of evidence†	9	State the process for selecting sources of evidence (i.e., screening and eligibility) included in the scoping review.	5-6
Data charting process‡	10	Describe the methods of charting data from the included sources of evidence (e.g., calibrated forms or forms that have been tested by the team before their use, and whether data charting was done independently or in duplicate) and any processes for obtaining and confirming data from investigators.	5-6
Data items	11	List and define all variables for which data were sought and any assumptions and simplifications made.	5-6
Critical appraisal of individual sources of evidence§	12	If done, provide a rationale for conducting a critical appraisal of included sources of evidence; describe the methods used and how this information was used in any data synthesis (if appropriate).	Not applicable
Synthesis of results	13	Describe the methods of handling and summarizing the data that were charted.	5-6
**RESULTS**			
Selection of sources of evidence	14	Give numbers of sources of evidence screened, assessed for eligibility, and included in the review, with reasons for exclusions at each stage, ideally using a flow diagram.	6
Characteristics of sources of evidence	15	For each source of evidence, present characteristics for which data were charted and provide the citations.	Table S5
Critical appraisal within sources of evidence	16	If done, present data on critical appraisal of included sources of evidence (see item 12).	Not applicable
Results of individual sources of evidence	17	For each included source of evidence, present the relevant data that were charted that relate to the review questions and objectives.	Table S5
Synthesis of results	18	Summarize and/or present the charting results as they relate to the review questions and objectives.	9-13
**DISCUSSION**			
Summary of evidence	19	Summarize the main results (including an overview of concepts, themes, and types of evidence available), link to the review questions and	14-16

SECTION	ITEM	PRISMA-ScR CHECKLIST ITEM	REPORTED ON PAGE #
		objectives, and consider the relevance to key groups.	
Limitations	20	Discuss the limitations of the scoping review process.	16
Conclusions	21	Provide a general interpretation of the results with respect to the review questions and objectives, as well as potential implications and/or next steps.	17
**FUNDING**			
Funding	22	Describe sources of funding for the included sources of evidence, as well as sources of funding for the scoping review. Describe the role of the funders of the scoping review.	17

References

1. Stratil, J.M., et al., *Development of an overarching framework for anticipating and assessing adverse and other unintended consequences of public health interventions (CONSEQUENT): A best-fit framework synthesis.* BMJ Public Health, 2024. **2**(1): p. e000209.
2. Al-Khudairy, L., et al., *Evidence and methods required to evaluate the impact for patients who use social prescribing: a rapid systematic review and qualitative interviews.* Health and Social Care Delivery Research, 2022. **10**(29).
3. Aughterson, H., L. Baxter, and D. Fancourt, *Social prescribing for individuals with mental health problems: a qualitative study of barriers and enablers experienced by general practitioners.* BMC Fam Pract, 2020. **21**(1): p. 194.
4. Trust, A.W., *Wellbeing with nature evaluation report: Improving people's health and wellbeing, whilst caring for the natural environment and deepening their connection to nature*. 2021.
5. Baker, K. and A. Irving, *Co-producing Approaches to the Management of Dementia through Social Prescribing.* Social Policy & Administration, 2016. **50**(3): p. 379-397.
6. Beardmore, A., *Working in social prescribing services: a qualitative study.* J Health Organ Manag, 2019. **34**(1): p. 40-52.
7. Bertotti, M., C. Frostick, and O. Temirov, *An Evaluation of Social Prescribing in the London Borough of Redbridge: Final Evaluation Report*. 2020, Institute for Health and Human Development, University of East London.
8. Bertotti, M., et al., *The Social Prescribing Service in the London Borough of Waltham Forest: Final Evaluation Report*. 2017, Institute for Health and Human Development, University of East London.
9. Bertotti, M., et al., *A realist evaluation of social prescribing: an exploration into the context and mechanisms underpinning a pathway linking primary care with the voluntary sector.* Prim Health Care Res Dev, 2018. **19**(3): p. 232-245.
10. Blickem, C., et al., *Linking people with long-term health conditions to healthy community activities: development of Patient-Led Assessment for Network Support (PLANS).* Health Expect, 2013. **16**(3): p. e48-59.
11. Brandling, J. and W. House, *Investigation into the feasibility of a social prescribing service in primary care: a pilot project*. 2007, University of Bath and Bath and North East Somerset NHS Primary Care Trust: Bath, U. K.
12. Brandling, J. and W. House, *Social prescribing in general practice: Adding meaning to medicine.* British Journal of General Practice, 2009. **59**(563): p. 454-456.
13. Bu, F., et al., *Equal, equitable or exacerbating inequalities? Patterns and predictors of social prescribing referrals in 160,128 UK patients.* medRxiv, 2024.
14. Bybee, S.G., et al., *A Secondary Data Analysis of Technology Access as a Determinant of Health and Impediment in Social Needs Screening and Referral Processes.* AJPM Focus, 2024. **3**(2): p. 100189.
15. Bywaters, P., et al., *Good intentions, increased inequities: Developing social care services in Emergency Departments in the UK.* Health and Social Care in the Community, 2011. **19**(5): p. 460-467.
16. Carnes, D., et al., *City and Hackney Social Prescribing Service: Evaluation Report*. 2015.
17. Carnes, D., et al., *The impact of a social prescribing service on patients in primary care: a mixed methods evaluation.* BMC Health Serv Res, 2017. **17**(1): p. 835.

18. Christofides, N. and R. Jewkes, *Acceptability of universal screening for intimate partner violence in voluntary HIV testing and counseling services in South Africa and service implications.* AIDS Care, 2010. **22**(3): p. 279-85.
19. Dayson, C. and E. Batty, *Social prescribing and the value of small providers: Evidence from the evaluation of the Rotherham social prescribing service*. 2020, Sheffield: Centre for Regional Economic and Social Research, Sheffield Hallam University.
20. Dayson, C. and C. Damm, *Evaluation of the Rotherham Social Prescribing Service for Long Term Conditions*. 2020, CRESR, Sheffield Hallam University.
21. Dayson, C., N. Bashir, and S. Pearson, *From dependence to independence: Emerging lessons from the Rotherham Social Prescribing project*. 2013.
22. Dayson, C., A. Fraser, and T. Lowe, *A Comparative Analysis of Social Impact Bond and Conventional Financing Approaches to Health Service Commissioning in England: The Case of Social Prescribing.* Journal of Comparative Policy Analysis: Research and Practice, 2019. **22**(2): p. 153-169.
23. Dickens, A.P., et al., *An evaluation of the effectiveness of a community mentoring service for socially isolated older people: a controlled trial.* BMC Public Health, 2011. **11**(1): p. 218.
24. Din, N.U., et al., *Health professionals' perspectives on exercise referral and physical activity promotion in primary care: Findings from a process evaluation of the National Exercise Referral Scheme in Wales.* Health Educ J, 2015. **74**(6): p. 743-757.
25. Ell, K., et al., *Working with Bilingual Community Health Worker Promotoras to Improve Depression and Self-Care among Latino Patients with Long-Term Health Problems.* 2018.
26. Elston, J., et al., *Does a social prescribing 'holistic' link-worker for older people with complex, multimorbidity improve well-being and frailty and reduce health and social care use and costs? A 12-month before-and-after evaluation.* Prim Health Care Res Dev, 2019. **20**: p. e135.
27. Farenden, C., *Community Navigation in Brighton & Hove Evaluation of a social prescribing pilot*. 2015, Brighton & Hove Impetus.
28. Faulkner, M., *Supporting the psychosocial needs of patients in general practice: the role of a voluntary referral service.* Patient Educ Couns, 2004. **52**(1): p. 41-6.
29. Fixsen, A. and S. Barrett, *Challenges and Approaches to Green Social Prescribing During and in the Aftermath of COVID-19: A Qualitative Study.* Front Psychol, 2022. **13**(101550902): p. 861107.
30. Fixsen, A., S. Barrett, and M. Shimonovich, *Weathering the storm: A qualitative study of social prescribing in urban and rural Scotland during the COVID-19 pandemic.* SAGE Open Med, 2021. **9**: p. 20503121211029187.
31. Foster, A., et al., *Impact of social prescribing to address loneliness: A mixed methods evaluation of a national social prescribing programme.* Health Soc Care Community, 2021. **29**(5): p. 1439-1449.
32. Frerichs, J., et al., *Influences on participation in a programme addressing loneliness among people with depression and anxiety: findings from the Community Navigator Study.* BMC Psychiatry, 2020. **20**(1): p. 565.
33. Frostick, C. and M. Bertotti, *The frontline of social prescribing - How do we ensure Link Workers can work safely and effectively within primary care?* Chronic Illn, 2021. **17**(4): p. 404-415.
34. Fullwood, Y., *Blended evaluation of Phase 2 of the Age UK Personalised Integrated Care Programme*. 2018, Understanding Value Ltd.

35. Galvin, K., A. Sharples, and D. Jackson, *Citizens Advice Bureaux in general practice: an illuminative evaluation.* Health Soc Care Community, 2000. **8**(4): p. 277-282.
36. Galway, K., et al., *Adapting Digital Social Prescribing for Suicide Bereavement Support: The Findings of a Consultation Exercise to Explore the Acceptability of Implementing Digital Social Prescribing within an Existing Postvention Service.* Int J Environ Res Public Health, 2019. **16**(22).
37. Garside, R., et al., *Therapeutic Nature: Nature-Based Social Prescribing for Diagnosed Mental Health Conditions in the UK. Final Report for DEFRA*. 2020.
38. Gibson, K., T.M. Pollard, and S. Moffatt, *Social prescribing and classed inequality: A journey of upward health mobility?* Soc Sci Med, 2021. **280**: p. 114037.
39. Giebel, C., N. Morley, and A. Komuravelli, *A socially prescribed community service for people living with dementia and family carers and its long-term effects on well-being.* Health Soc Care Community, 2021. **29**(6): p. 1852-1857.
40. Grant, C., et al., *A randomised controlled trial and economic evaluation of a referrals facilitator between primary care and the voluntary sector.* BMJ, 2000. **320**(7232): p. 419-23.
41. Griffith, B., et al., *Constituting link working through choice and care: An ethnographic account of front-line social prescribing.* Sociol Health Illn, 2023. **45**(2): p. 279-297.
42. Griffiths, C., H. Jiang, and K. Walker, *Social Prescribing: Link Workers' Perspectives on Service Delivery.* Open Journal of Social Sciences, 2023. **11**(05): p. 63-80.
43. Hamlin, M.J., et al., *Long-term effectiveness of the New Zealand Green Prescription primary health care exercise initiative.* Public Health, 2016. **140**: p. 102-108.
44. Hanlon, P., et al., *Does Self-Determination Theory help explain the impact of social prescribing? A qualitative analysis of patients' experiences of the Glasgow 'Deep-End' Community Links Worker Intervention.* Chronic Illn, 2021. **17**(3): p. 173-188.
45. Hazeldine, E., et al., *Link worker perspectives of early implementation of social prescribing: A 'Researcher-in-Residence' study.* Health Soc Care Community, 2021. **29**(6): p. 1844-1851.
46. Heijnders, M.L. and J.J. Meijs, *'Welzijn op Recept' (Social Prescribing): a helping hand in re-establishing social contacts - an explorative qualitative study.* Prim Health Care Res Dev, 2018. **19**(3): p. 223-231.
47. Holding, E., et al., *Connecting communities: A qualitative investigation of the challenges in delivering a national social prescribing service to reduce loneliness.* Health & social care in the community, 2020. **28**(5): p. 1535-1543.
48. Unit, I., *Wigan Community Link Worker Service Evaluation*. 2016.
49. Isaacs, A.J., et al., *Exercise Evaluation Randomised Trial (EXERT): a randomised trial comparing GP referral for leisure centre-based exercise, community-based walking and advice only.* Health Technol Assess, 2007. **11**(10): p. 1-165, iii-iv.
50. Islam, N., *Social Prescribing Service Bromley by Bow Centre Annual Report: April 2018 - March 2019*. 2019.
51. Islington, H., *Social prescribing and navigation services in Islington*. 2019.
52. Jones, C. and M. Lynch, *Spice time credits social prescribing pilot evaluation*. 2019.

53. Kellezi, B., et al., *The social cure of social prescribing: a mixed-methods study on the benefits of social connectedness on quality and effectiveness of care provision.* BMJ Open, 2019. **9**(11): p. e033137.
54. Kellezi, B., M. Bowe, and J. Wakefield, *Public Health England Social Prescribing Approaches for Migrants: Call for Evidence Submission (unpublished results)*. 2020.
55. Council;, K.C.S. and N.W.L.C. Commissioning, *Self-Care Social Prescribing: Social Return on Investment*. 2018, Envoy Partnership.
56. Khan, K., et al., *The feasibility of identifying health inequalities in social prescribing referrals and declines using primary care patient records.* NIHR Open Res, 2023. **3**(9918333281906676): p. 1.
57. Kharicha, K., et al., *What do older people experiencing loneliness think about primary care or community based interventions to reduce loneliness? A qualitative study in England.* Health Soc Care Community, 2017. **25**(6): p. 1733-1742.
58. Kiely, B., et al., *Primary care-based link workers providing social prescribing to improve health and social care outcomes for people with multimorbidity in socially deprived areas (the LinkMM trial): Pilot study for a pragmatic randomised controlled trial.* J Multimorb Comorb, 2021. **11**(9918333280706676): p. 26335565211017781.
59. Kimberlee, R., *Gloucestershire clinical commissioning group's social prescribing service: Evaluation report*. 2016.
60. Longwill, A., *Independent Evaluation of Hackney WellFamily Service*. 2014, Improving Health and Wellbeing UK.
61. Loo, S.S., *Considering the impact of social risk screening and referral interventions on adults in the safety-net: A mixed methods approach to health system perspectives*. 2023, Boston University School of Public Health.
62. Lowe, T., et al., *The institutional work of creating and implementing Social Impact Bonds.* Policy & Politics, 2019. **47**(2): p. 353-369.
63. McHale, S., et al., *Green Health Partnerships in Scotland; Pathways for Social Prescribing and Physical Activity Referral.* Int J Environ Res Public Health, 2020. **17**(18): p. 6823.
64. McLeish, J. and M. Redshaw, *Peer support during pregnancy and early parenthood: a qualitative study of models and perceptions.* BMC Pregnancy Childbirth, 2015. **15**: p. 257.
65. McLeish, J. and M. Redshaw, *'We have beaten HIV a bit': a qualitative study of experiences of peer support during pregnancy with an HIV Mentor Mother project in England.* BMJ Open, 2016. **6**(6): p. e011499.
66. McLeish, J. and M. Redshaw, *"I didn't think we'd be dealing with stuff like this": A qualitative study of volunteer support for very disadvantaged pregnant women and new mothers.* Midwifery, 2017. **45**: p. 36-43.
67. Mistry, S.K., et al., *Feasibility and acceptability of involving bilingual community navigators to improve access to health and social care services in general practice setting of Australia.* BMC Health Serv Res, 2023. **23**(1): p. 476.
68. Moffatt, S., et al., *Impact of a social prescribing intervention in North East England on adults with type 2 diabetes: the SPRING_NE multimethod study.* Public Health Res, 2023. **11**(2): p. 1-185.
69. Moore, C., et al., *"Winging It": An Exploration of the Self-Perceived Professional Identity of Social Prescribing Link Workers.* Health & Social Care in the Community, 2023. **2023**: p. 1-8.

70. Morris, S.L., et al., *Social prescribing during the COVID-19 pandemic: a qualitative study of service providers' and clients' experiences.* BMC health services research, 2022. **22**(1): p. 258.
71. Workers, N.A.o.L., *Getting to know the link worker workforce: Understanding link workers knowledge, skills, experiences and support needs*. 2019.
72. (NHS), N.H.S., *Social prescribing and community-based support. Summary guide*. 2020.
73. O'Brien, L., M. Townsend, and M. Ebden, *'Doing Something Positive': Volunteers' Experiences of the Well-Being Benefits Derived from Practical Conservation Activities in Nature.* Voluntas, 2010. **21**(4): p. 525-545.
74. Palmer, D. and P.N. Sango, *Social prescribing in Bexley: pilot evaluation report*. 2017.
75. Patel, S., et al., *Opportunities and Challenges for Digital Social Prescribing in Mental Health: Questionnaire Study.* J Med Internet Res, 2021. **23**(3): p. e17438.
76. Pescheny, J., G. Randhawa, and Y. Pappas, *Patient uptake and adherence to social prescribing: a qualitative study.* BJGP Open, 2018. **2**(3): p. bjgpopen18X101598.
77. Pescheny, J.V., et al., *The impact of the Luton social prescribing programme on mental well-being: a quantitative before-and-after study.* J Public Health (Oxf), 2021. **43**(1): p. e69-e76.
78. Pollard, T., et al., *Implementation and impact of a social prescribing intervention: an ethnographic exploration.* Br J Gen Pract, 2023. **73**(735): p. e789-e797.
79. Pons-Vigues, M., et al., *Qualitative evaluation of a complex intervention to implement health promotion activities according to healthcare attendees and health professionals: EIRA study (phase II).* BMJ Open, 2019. **9**(3): p. e023872.
80. Poole, R. and P. Huxley, *Social prescribing: an inadequate response to the degradation of social care in mental health.* BJPsych Bull, 2024. **48**(1): p. 30-33.
81. Rathbone, A.P., et al., *"You don't get side effects from social prescribing"-A qualitative study exploring community pharmacists' attitudes to social prescribing.* PLoS ONE, 2024. **19**(5): p. e0301076.
82. Rhodes, J. and S. Bell, *"It sounded a lot simpler on the job description": A qualitative study exploring the role of social prescribing link workers and their training and support needs (2020).* Health Soc Care Community, 2021. **29**(6): p. e338-e347.
83. Robinson, J.M., et al., *Let Nature Be Thy Medicine: A Socioecological Exploration of Green Prescribing in the UK.* Int J Environ Res Public Health, 2020. **17**(10): p. 3460.
84. Rowe, C., N. Nia Wildblood, and F. S., *Social Prescribing Impact Report 2019-2020*. 2020.
85. Scarpetti, G., et al., *A comparison of social prescribing approaches across twelve high-income countries.* Health Policy, 2024. **142**: p. 104992.
86. Schmidt, M., et al., *Which factors engage women in deprived neighbourhoods to participate in exercise referral schemes?* BMC Public Health, 2008. **8**: p. 371.
87. Simpson, S., et al., *Supporting access to activities to enhance well-being and reduce social isolation in people living with motor neurone disease.* Health Soc Care Community, 2020. **28**(6): p. 2282-2289.
88. Skivington, K., et al., *Delivering a primary care-based social prescribing initiative: a qualitative study of the benefits and challenges.* Br J Gen Pract, 2018. **68**(672): p. e487-e494.

89. South, J., et al., *Can social prescribing provide the missing link?* Primary Health Care Research & Development, 2008. **9**(04): p. 310-318.
90. Southby, K. and M. Gamsu, *Factors affecting general practice collaboration with voluntary and community sector organisations.* Health Soc Care Community, 2018. **26**(3): p. e360-e369.
91. Strachan, G., G.D. Wright, and E. Hancock, *An evaluation of a community health intervention programme aimed at improving health and wellbeing.* Health Education Journal, 2007. **66**(3): p. 277-285.
92. Stuart, A., et al., *'Oh no, not a group!' The factors that lonely or isolated people report as barriers to joining groups for health and well-being.* Br J Health Psychol, 2022. **27**(1): p. 179-193.
93. Sumner, R.C., et al., *Factors associated with attendance, engagement and wellbeing change in an arts on prescription intervention.* J Public Health, 2020. **42**(1): p. e88-e95.
94. Todd, C., *Exploring the role of museums for socially isolated older people*. 2017, Canterbury Christ Church University.
95. Tran, L., *Evaluation of a Chinese Mental Health Advocacy and Support Project*. 2009, Chinese National Healthy Living Centre.
96. Vogelpoel, N. and K. Jarrold, *Social prescription and the role of participatory arts programmes for older people with sensory impairments.* Journal of Integrated Care, 2014. **22**(2): p. 39-50.
97. Westlake, D., et al., *"She's Been a Rock": The Function and Importance of "Holding" by Social Prescribing Link Workers in Primary Care in England—Findings from a Realist Evaluation.* Health & Social Care in the Community, 2024. **2024**(1): p. 16.
98. White, J. and K. Kinsella, *An Evaluation of Social Prescribing Health Trainers in South and West Bradford*. 2010, Leeds Metropolitan University.
99. Whitelaw, S., et al., *Developing and implementing a social prescribing initiative in primary care: insights into the possibility of normalisation and sustainability from a UK case study.* Prim Health Care Res Dev, 2017. **18**(2): p. 112-121.
100. Wildman, J.M., et al., *Link workers' perspectives on factors enabling and preventing client engagement with social prescribing.* Health Soc Care Community, 2019. **27**(4): p. 991-998.
101. Wildman, J.M., et al., *Service-users' perspectives of link worker social prescribing: a qualitative follow-up study.* BMC Public Health, 2019. **19**(1): p. 98.
102. Wilson, N., *Branching out: greenspace and conservation on referral*. 2009.
103. Woodall, J., et al., *Understanding the effectiveness and mechanisms of a social prescribing service: a mixed method analysis.* BMC Health Serv Res, 2018. **18**(1): p. 604.
104. Wormald, H. and L. Ingle, *GP exercise referral schemes: Improving the patient's experience.* Health Education Journal, 2004. **63**(4): p. 362-373.
105. Wormald, H., et al., *Participants' perceptions of a lifestyle approach to promoting physical activity: targeting deprived communities in Kingston-upon-Hull.* BMC Public Health, 2006. **6**: p. 202.
106. Yichao, H., et al., *Negotiating Complexity: Challenges to Implementing Community-Led Nature-Based Solutions in England Pre- and Post-COVID-19.* International Journal of Environmental Research and Public Health, 2022. **19**(22): p. 14906.

107. Tricco, A.C., et al., *PRISMA Extension for Scoping Reviews (PRISMA-SCR): Checklist and explanation.* Annals of Internal Medicine, 2018. **169**(7): p. 467-473.
